# The impact of family capital differences on music majors' academic achievement: an empirical analysis based on structural equation model

**DOI:** 10.3389/fpsyg.2026.1856263

**Published:** 2026-07-31

**Authors:** Shuyun Li

**Affiliations:** School of Performing Arts, Geely University of China, Chengdu, China

**Keywords:** family capital, moderated mediation, multidimensional academic achievement, multigroup structural equation modeling, music higher education

## Abstract

This study examines how family capital differences affect music majors' academic achievement, an underexplored educational equity issue. Using Bourdieu's and Bandura's theories, we built a multi-path mediation structural equation model with three family capitals (economic, cultural, social) as predictors, learning engagement and self-efficacy as parallel mediators, and multidimensional achievement as outcome. Based on 952 music undergraduates from eight Chinese universities across eastern, central, and western regions, we used CFA, bootstrap mediation, and multigroup path comparison including a 2 × 2 crossed analysis. The dependent variable was measured through a multi-source design combining faculty rating [ICC (2, *k*) = 0.86], official course-grade records, and artistic-practice evaluation, partially decoupling it from self-report. The results show that (a) cultural capital is the strongest predictor of multidimensional academic achievement (β = 0.19, *p* < 0.001), followed by the direct effect of economic capital (β = 0.12, *p* = 0.004), while the effect of social capital qualifies as indirect-only mediation—the direct path within the full structural model is non-significant (β = 0.08, *p* = 0.060) and both indirect paths through learning engagement and professional self-efficacy are significant; (b) learning engagement and professional self-efficacy play significant parallel mediating roles between family capital and academic achievement; (c) institution type and student origin moderate the capital-to-mediator pathways in directions consistent with theory—the cultural capital → professional self-efficacy path is stronger in key universities than in regular universities (Δβ = 0.14, *p* = 0.009), and the economic capital → learning engagement path is stronger among rural students than urban students (Δβ = 0.14, *p* = 0.015)—while mediator-to-outcome paths show no between-group variation. The structural estimates remained stable under covariate-controlled and reverse-direction robustness checks. This study identifies a domain-specific inversion of the family capital hierarchy in art higher education (traced to the primacy of embodied cultural capital, upstream selection through specialized music admission, and the role of aesthetic taste as an evaluative criterion), develops a multi-source multidimensional achievement measurement tool for music majors, and reveals a stage-specific moderated mediation mechanism that provides empirical and policy references for promoting equity in music higher education.

## 1 Introduction

Music education occupies a unique position in the global higher education system. It not only requires students to have solid theoretical literacy, but also sets high standards for practical skills. Different from the general disciplines, the academic achievement of music majors not only depends on the learning input in the classroom, but also is deeply influenced by the material conditions and cultural atmosphere provided by the family. The acquisition of resources such as the purchase of musical instruments, extracurricular professional training, and concert viewing experience is often closely related to the economic strength and cultural tendency of families. This connection is increasingly prominent in the context of the massification of higher education, as growing numbers of students from diverse backgrounds enter the music major, but there are significant differences in the family capital they carry. Bourdieu's capital theory (Bourdieu, 1986) divides family capital into three dimensions: economic capital, cultural capital and social capital, which provides a solid analytical framework for understanding this difference. Family economic capital determines whether students can afford high-quality professional training. Cultural capital shapes students' cognitive depth and aesthetic orientation of music, while social capital affects students' access to professional development opportunities. As educational equity attracts increasing attention, it is of considerable theoretical and practical significance to systematically investigate how family capital differences affect the academic performance of music majors.

In recent years, the research on the relationship between family capital and academic achievement has made substantial progress. A cross-border comparative study based on PISA 2022 data shows that family cultural capital has a significant positive predictive effect on students' cognitive performance, and academic engagement and academic self-efficacy play a partial mediating role (Djekourmane et al., 2025). In the Chinese context, institutionalized cultural capital and objectified cultural capital have significantly promoted the academic performance of middle school students, but this effect is heterogeneous between different gender groups (Jin et al., 2024). A study using structural equation model to analyze Czech PISA data found that family highbrow cultural resources and reading interest indirectly affect academic performance through non-cognitive ability, while the mediating effect of the teacher–student relationship is negligible. This conclusion calls into question the core hypothesis of Bourdieu's cultural reproduction theory (Raudenšká, 2022). The analysis of Chinese youth groups reveals that the effect path of family social capital and cultural capital on academic effort, educational expectation and academic achievement is not consistent, and the two types of capital play a role through different channels (Tan and Fang, 2023b). The research on the influence mechanism of family cultural capital on Chinese junior high school students' academic achievement found that parents' cultural radiation and parenting behavior can help their children acquire the cognitive and non-cognitive abilities required for academic success (Yu et al., 2022). In the field of music education, studies have examined the participation and sustainability of American high school students' elective music ensemble courses. The results show that family socio-economic status, academic performance and participation in off-campus artistic activities are the key factors to predict the sustainability of students' music learning (Elpus and Abril, 2025). Another study incorporated the habitus and field in Bourdieu's practice theory into the analysis framework, built a chain mediation model from family cultural capital to academic achievement, and revealed that cultural capital did not directly promote academic performance, but indirectly played a role by shaping individual habitus and interacting with the school field (Jin et al., 2025). A systematic literature review pointed out that the concept of cultural capital in the Chinese context has local characteristics, and the dimensions of shadow education participation, parent–child reading and English ability show high explanatory power in the prediction of academic achievement (Xie and Hutagalung, 2025). In the research of music education in colleges and universities, some scholars based on self-determination theory discussed the influence path of music listening willingness and music engagement on the achievement of college music students (Wang and Huang, 2024). However, most of the above studies focus on the basic education stage or comprehensive disciplines, and the empirical analysis for art majors, especially music majors, is still scarce. The academic achievement evaluation system of music majors includes multiple dimensions such as performance skills, theoretical examination, and stage practice. Its formation mechanism is essentially different from that of general disciplines, and the existing model is difficult to be directly applied.

Based on the above research status, this paper selects music majors as the research object, measures the difference of family capital from the three dimensions of economic capital, cultural capital and social capital, and introduces learning engagement and professional self-efficacy as mediating variables to construct a structural equation model for empirical test. The innovations of this study are reflected in three aspects.

First, this study does not merely transfer Bourdieu's family capital framework to a new population label, but identifies a domain-specific inversion of the capital hierarchy in art higher education. In basic education research, family economic capital typically exerts the dominant family-level effect on academic outcomes (Yigiter, 2025; Wang and Wu, 2023). In the music major context examined here, cultural capital surpasses economic capital in direct effect magnitude (β = 0.19 vs. β = 0.12), and social capital exerts only fully mediated effects with a non-significant direct path. This inverted hierarchy challenges the implicit assumption that resource-intensive art training is primarily driven by economic affordability, and points to embodied aesthetic dispositions as the more decisive family-level resource in art higher education—a mechanism-level finding that has not been documented in the existing family capital literature. Rather than leaving this inversion as a bare empirical fact, the present study traces it to three complementary mechanisms—the primacy of embodied cultural capital in music attainment, the upstream filtering imposed by China's specialized music entrance examination, and the role of cultivated aesthetic taste as part of the instructional content and evaluation criteria of arts training—which are developed in Section 5.1.

Second, this study develops and validates a multidimensional academic achievement scale that is specifically adapted to the structural features of music undergraduate training. The scale integrates performance skill evaluation (rated by faculty using a standardized rubric with inter-rater reliability assessment), theoretical achievement (obtained from official course-grade records), and artistic practice performance (composed of competition awards and stage performance evaluation). Unlike GPA-based or single-test-score measures that dominate the existing literature (Jin et al., 2024), this multi-source measurement design partially separates the dependent variables from self-report and reduces common method bias risk by design rather than only by statistical control.

Third, this study extends moderated mediation analysis to the family capital field by simultaneously testing two theoretically grounded moderators—institution type and student origin—on the capital-to-mediator pathways. The analysis identifies that the moderating effects of family background are concentrated at the capital-to-mediator stage rather than the mediator-to-outcome stage, suggesting that the conversion of engagement and self-efficacy into achievement operates as a domain-general process while the activation of family resources is contingent on institutional and geographic conditions. This stage-specific moderation finding offers a refinement of moderated mediation theory that is empirically grounded rather than *post hoc*.

This study aims to answer a core question: through what mechanisms do family capital differences affect the academic achievement of music majors? The answer to this question can not only enrich the application of Bourdieu's capital theory in the field of art higher education, but also provide operable policy references for promoting the equity of music higher education.

## 2 Literature review

### 2.1 The evolution of family capital theory and its measurement dilemma

Bourdieu's capital theory (Bourdieu, 1986) constitutes a core analytical framework for understanding the relationship between family background and educational inequality. The theory divides family capital into three forms: economic capital, cultural capital and social capital, and emphasizes the dynamic relationship among the three. Bourdieu (1986, pp. 243–248) further subdivides cultural capital into three states: embodied (long-lasting dispositions of the mind and body), objectified (cultural goods such as pictures, books, instruments, and machines), and institutionalized (educational qualifications). This tripartite classification, together with the cultural reproduction mechanism formalized in Bourdieu and Passeron (1990), provides an important operational direction for subsequent empirical research (de Moll et al., 2024). Recent work has further scrutinized the cultural-reproduction account by disentangling genetic from social pathways of cognitive transmission (Wolfram et al., 2024) and by re-examining cultural reproduction theory in the Chinese adolescent context (Xie and He, 2026). The aesthetic-disposition argument developed in Bourdieu (1984) is particularly relevant for music education research, because it specifies that the recognition and appreciation of cultivated cultural forms is itself transmitted through family socialization rather than acquired through formal schooling alone. The meta-analysis of the relationship between cultural capital and reading performance found that cultural capital has a stable positive predictive effect on students' reading performance, but the effect quantity shows significant heterogeneity in different countries and education systems (Jheng et al., 2023). This finding implies that the concept of cultural capital in Bourdieu's theory faces adaptive challenges in the process of cross-cultural migration. Studies have identified three different types of academic habits among middle school students through latent class analysis—excellence, adaptation and alienation, revealing the non-linear correspondence between family capital structure and students' behavioral tendencies (Yigiter, 2025). The cross-border longitudinal study on the relationship between socio-economic status and academic achievement further shows that socio-economic status at the individual level and school level has a positive impact on academic performance, and the effect at the school level is more prominent (Wang and Wu, 2023). Across educational systems, cultural capital and family socioeconomic status remain robust predictors of school performance (Nunes and Andrade, 2024; Perry et al., 2022; Song et al., 2025). The problem is that most studies only focus on the single dimension of cultural capital, and lack consensus on the operational measurement of economic capital and social capital. Especially for the measurement of social capital, different researchers' selection of indicators varies greatly—some are measured by the frequency of parent–child interaction, and some are measured by the size of parents' social network. This operational difference directly weakens the comparability of research conclusions (Tan and Fang, 2023b). In the Chinese context, the continuous expansion of income inequality has led to structural changes in education investment behavior. Based on random class data of Chinese middle school students, studies have investigated the impact of economic inequality in the classroom on academic performance, and found that higher economic inequality in the classroom significantly reduced the test scores of students with low socio-economic status by weakening students' educational expectations and parents' participation (Jiang, 2024). In the field of music education, the conceptualization of family capital is more distinctive. Musical instrument ownership belongs to objectified cultural capital, parents' music literacy belongs to embodied cultural capital, and parents' formal music qualifications belong to institutionalized cultural capital, while the professional relationship between parents and music teachers falls under social capital (Bourdieu, 1986). Generic measurement tools cannot capture these discipline-specific forms of capital, which is the central reason this study develops a customized, music-specific operationalization of the three capital forms rather than importing an off-the-shelf scale.

### 2.2 Methodological bottleneck of the relationship between family capital and academic achievement

At the level of analytical methods, the research on the relationship between family capital and academic achievement has long relied on linear regression or single-mediator structural models, which is difficult to reveal the complex path mechanism when multidimensional capital acts simultaneously. Specifically, several representative studies illustrate this methodological limitation. Yu et al. (2022) examined family cultural capital and academic achievement in Chinese junior high school students using hierarchical regression, but did not test sequential mediation through both behavioral and psychological variables. Jin et al. (2024) employed a structural model in which cultural capital was linked to academic outcomes through a single non-cognitive mediator, omitting behavioral engagement from the pathway. Tan and Fang (2023b) tested the mediating role of family interaction patterns one variable at a time without modeling the simultaneous operation of behavioral and motivational mediators. These designs share a common feature: they cannot capture how behavioral and psychological mediators operate in parallel, which is exactly the gap that the present study targets. Although structural equation models have been adopted in some recent studies, two prominent problems arise in model design. On the one hand, most studies only test the direct effect or single mediation effect of family capital on academic achievement, ignoring the parallel mediating role of psychological and behavioral variables such as learning engagement and self-efficacy in the transmission path (Djekourmane et al., 2025). Although the structural equation model research using PISA data confirms the path that cultural capital indirectly affects academic achievement through non-cognitive ability, the mediation mechanism it examines is relatively simple and fails to cover variables at the behavioral level such as learning engagement (Raudenšká, 2022). In the field of music education, studies have found that music learning can indirectly improve academic performance by improving self-esteem and self-efficacy, which shows that adding psychological mediators between capital and achievement is theoretically reasonable (Gu et al., 2024). On the other hand, existing studies remain seriously inadequate in their examination of moderation effects. Grouping variables such as urban–rural differences, institutional levels, and gender may change the intensity and direction of the role of family capital, but empirical studies using multigroup comparative analysis remain rare (Jin et al., 2025). In the Chinese context, the urban–rural dual structure leads to essential differences in the composition of social capital and cultural capital between rural and urban families, which may lead to different fitting results of the same model in different groups (Tan and Fang, 2023a).

In rural China, the impact of family environment and parental participation on academic achievement even exceeds the direct effect of socio-economic status itself, which means that it is inappropriate to simply equate family capital with socio-economic status (Wang and Li, 2024). In terms of data sources, a large number of studies rely on PISA, CEPS and other large-scale survey data sets. Although the sample size is sufficient, these data are not designed for specific disciplines—for instance, Wang and Wu (2023) and Yang et al. (2022) both drew on PISA, whose academic achievement indicators mainly measure reading, mathematics and scientific literacy, none of which can reflect the performance of music majors in performance skills and artistic practice. The research on the relationship between academic self-efficacy and academic performance of music majors also reveals the limitations of the universal scale in the field of art (Gill et al., 2024). These methodological deficiencies indicate the need for a multi-path, multigroup structural equation model specifically designed for music education scenarios.

### 2.3 Limitations of the academic achievement evaluation system of art disciplines

The measurement of academic achievement is the cornerstone of empirical research, but in art disciplines, the limitations of the existing evaluation system are particularly prominent. Academic achievement in mainstream education research is usually measured by standardized test scores or GPA (Jin et al., 2024), which assumes that academic achievement is a one-dimensional construct. For music majors, this assumption clearly does not hold. The academic performance of music students covers many interrelated but different dimensions, such as performance or vocal music skills, music theoretical knowledge, solfeggio ability, stage practice experience and artistic creativity. From the perspective of self-determination theory, studies have explored the impact of music listening willingness and music engagement on the development and achievement of college music students, and found that music aesthetic experience plays a significant mediating role (Wang and Huang, 2024). This shows that the academic achievement of music major not only includes quantifiable skill level, but also involves subjective psychological experience such as aesthetic feeling. In the field of self-efficacy research, music performance self-efficacy is considered to be one of the strongest indicators to predict the quality of performance, which is essentially different from the general academic self-efficacy in terms of its conceptual connotation and measurement methods (Zhang, 2025). The domain-specificity of academic self-efficacy has likewise been emphasized in recent evidence (Hinduja et al., 2024).

The research on the relationship between metacognition and performance achievements of Chinese college music students also shows that cognitive self-confidence and thinking control ability have a significant impact on music performance output (Jiang and Tong, 2025). The systematic literature review of music students' self-esteem further confirmed that music teaching can improve students' self-esteem level through the mechanisms of mastering experience and social persuasion, and self-esteem is closely related to academic achievement and mental health (Wang, 2025). In terms of the relationship between socio-economic status and music education participation, relevant studies have confirmed that students from families with high socio-economic status have significantly higher participation rate and persistence rate in music courses (Elpus and Abril, 2025), but these studies focus on “whether to participate” rather than “the level of achievement after participation”.

For college music students, the joint mechanism of mindfulness, subjective well-being and music engagement on academic performance has been preliminarily explored (He et al., 2024), but whether the relationship between well-being and academic achievement is established in the music professional group, and whether family capital indirectly affects academic performance through the path of well-being remain unresolved. In the music domain specifically, music empathy and music engagement have been linked to students' subjective well-being (Tu and Fu, 2024). This open question is sharpened by a broader body of evidence in which academic achievement and student well-being are treated as reciprocally entwined rather than independent: multidimensional well-being and achievement can operate as a mutual trade-off (Ling et al., 2022), and academic performance is closely coupled with college students' subjective well-being (Zhai et al., 2026). Critically, however, this evidence rests almost entirely on general-discipline samples and one-dimensional achievement indices, leaving unexamined how such psychological coupling is shaped by family-level antecedents in a multidimensional, skill-based domain such as music—precisely the intersection the present study targets. Academic pressure faced by students in music education in Chinese colleges and universities has also attracted scholars' attention. Some studies have systematically evaluated the intervention strategies of music education by using fuzzy analytic hierarchy process and fuzzy TOPSIS method (Schunk and DiBenedetto, 2021). These findings converge on a central problem: the use of generic academic achievement measurement tools to evaluate the performance of music majors results in a serious loss of construct validity. How the academic achievement of music majors is affected by family capital differences has not been fully empirically answered.

### 2.4 Existing research gaps and theoretical model construction of this paper

Based on the above analysis, three core research gaps can be extracted. First, research applying family capital theory in music education is almost nonexistent. The existing research focuses on the general disciplines such as Chinese, mathematics and Science in the basic education stage (Djekourmane et al., 2025; Jin et al., 2024, 2025), and the coverage of higher education in the art category is seriously insufficient. Although transnational studies have revealed the general laws of family cultural capital affecting academic achievement (Wang and Wu, 2023), whether these laws can be applied to the highly specialized field of music education has not been tested. Second, measurement tools for academic achievement are not adapted to the characteristics of the music major. Generic indicators cannot effectively evaluate the key dimensions such as performance skills, stage performance and artistic creativity (Wang and Huang, 2024; Jiang and Tong, 2025). Third, the mediation mechanism and boundary conditions of family capital affecting academic achievement have not been systematically tested in the context of music education. Learning engagement and professional self-efficacy as mediating variables, as well as the boundary effect of college level and urban and rural background as moderating variables, lack empirical evidence from music-major samples.

**Figure 1:**
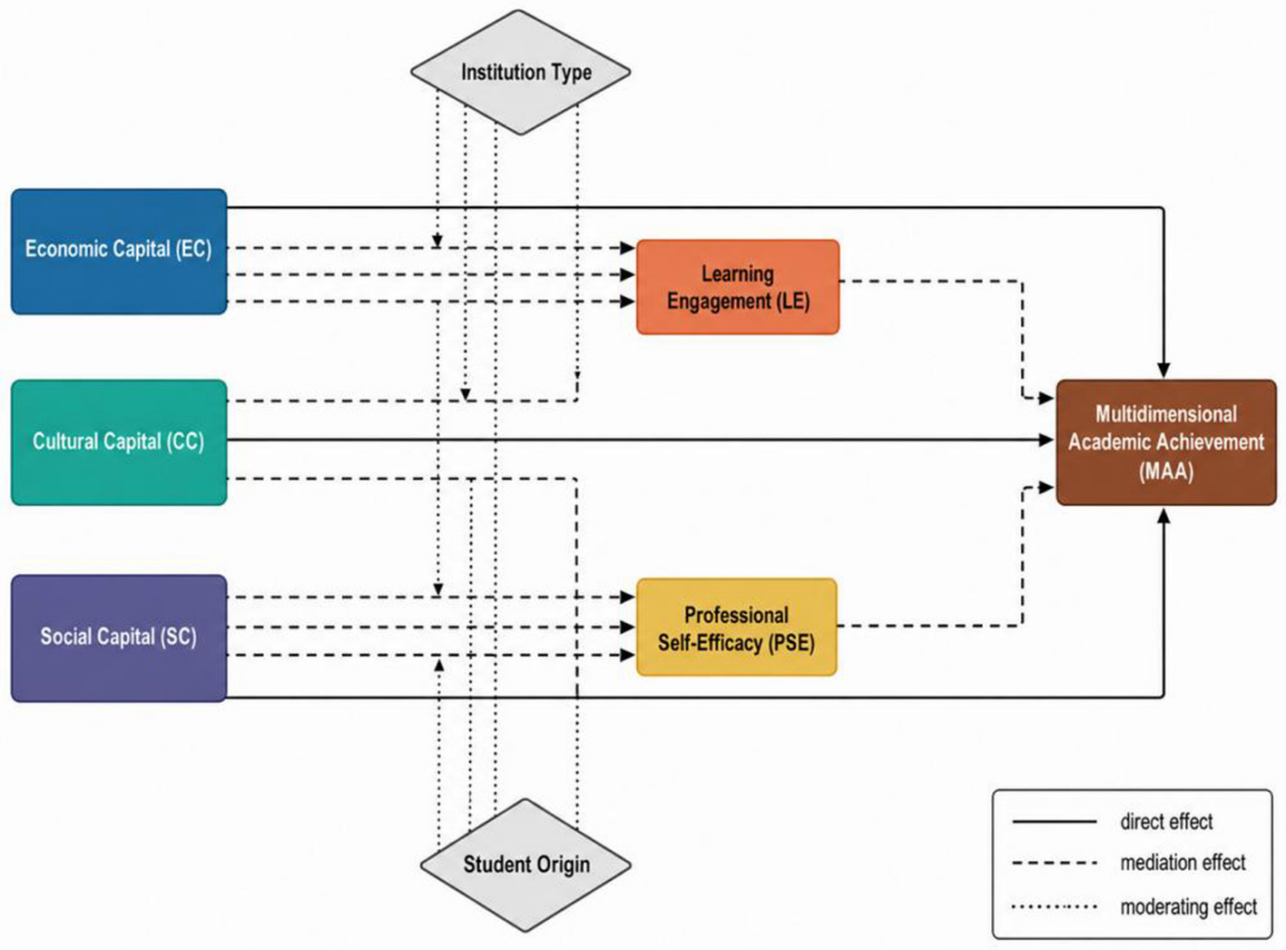
Theoretical model of the impact of family capital differences on the academic achievement of music majors.

Based on the above research gap, this paper constructs a multi-path mediation model (as shown in Figure 1). The model takes family economic capital, cultural capital and social capital as exogenous latent variables, multi-dimensional academic achievement of music major (performance skills, theoretical achievements, and artistic practice performance) as endogenous latent variables, learning engagement and professional self-efficacy as mediating variables, and college level and urban and rural background as moderating variables. In addition to the direct path, the model sets two mediation paths: family capital affects academic achievement through learning engagement, and family capital affects academic achievement through professional self-efficacy. The moderating variables are placed on the mediation pathways to test the difference of path coefficient between different groups. The model integrates Bourdieu's capital reproduction theory and Bandura's social cognition theory (Tan and Fang, 2023a) in theory, adopts multigroup structural equation model in method to estimate the differences between groups at the same time, and develops an academic achievement evaluation scale adapted to the characteristics of music major in measurement. The combination of the three constitutes the core contribution of this study which is different from the existing literature.

## 3 Research design

### 3.1 Theoretical model and research hypotheses

**Figure 2:**
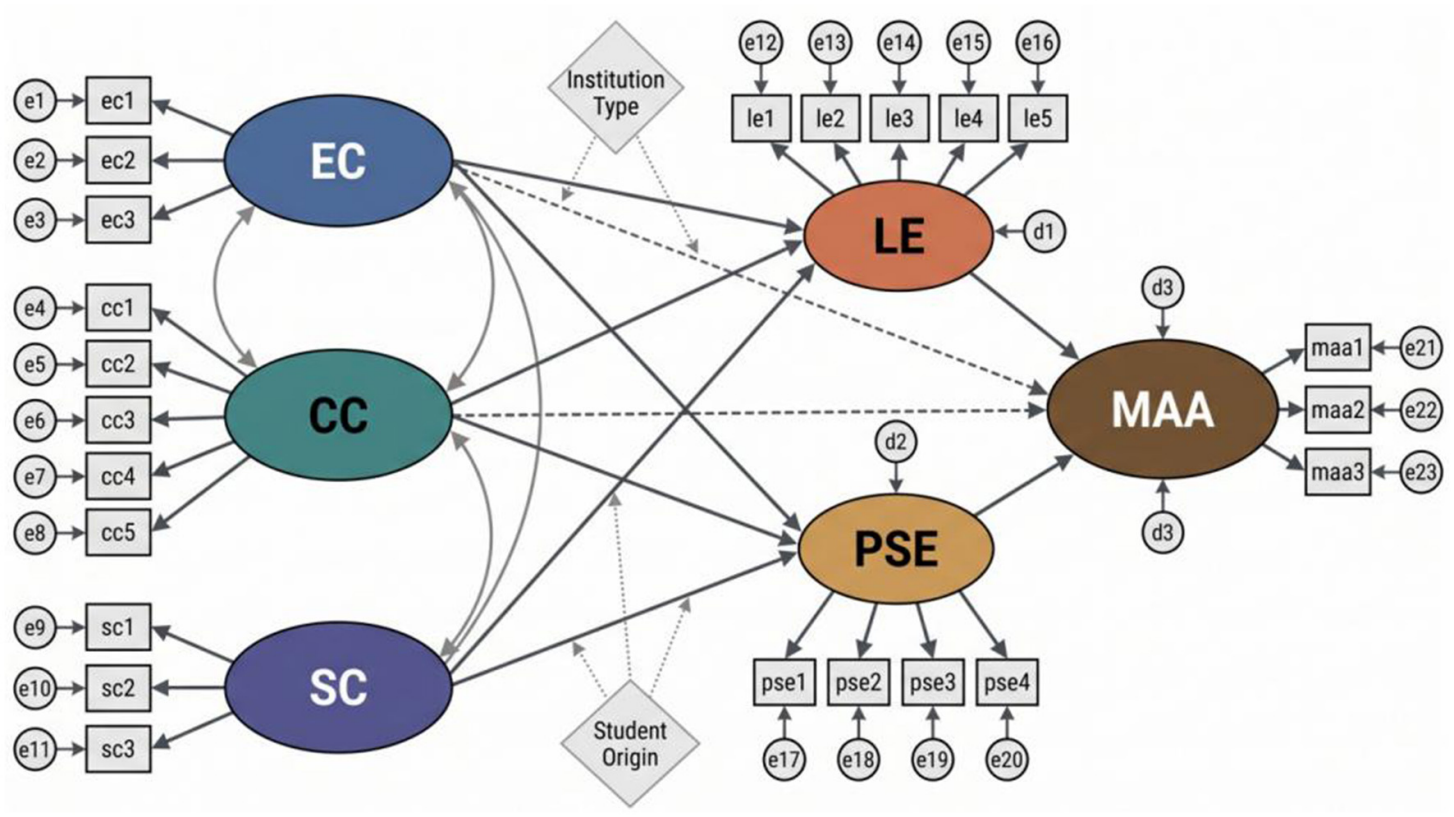
Structural equation path model of the impact of family capital differences on the academic achievement of music majors.

Based on the literature review and theoretical analysis in the second chapter, this study constructs a multi-path mediation model of family capital differences affecting the academic achievement of music majors. The core logic of the model includes three levels: the direct effect level—family economic capital, cultural capital and social capital have a direct impact on the multidimensional academic achievement of music majors respectively; the mediation effect level—three types of family capital indirectly affect academic achievement through two mediation paths of learning engagement and professional self-efficacy; the moderation effect level—the key coefficients in the mediation paths are moderated by institution type (key universities and regular universities) and student origin (urban and rural) as moderating variables. The complete theoretical model is shown in Figure 2.

Based on this model, the following hypotheses are proposed in this study.

**H1a, H1b, H1c:** Family economic capital, cultural capital and social capital each have a significant positive direct effect on music majors' multidimensional academic achievement.

**H2a, H2b, H2c:** Learning engagement plays a mediating role between three types of family capital (economic, cultural, social) and academic achievement, respectively.

**H3a, H3b, H3c:** Professional self-efficacy plays a mediating role between three types of family capital (economic, cultural, social) and academic achievement, respectively.

The two moderators examined below—institution type and student origin—are not chosen *ad hoc* but follow from Bourdieu's relational premise that the conversion of capital into outcomes depends on the field in which the capital is activated and on the structural position of the family that transmits it. Institution type operationalizes the field dimension: key institutions constitute a denser professional field—more frequent master classes, higher-caliber peers, and richer performance exposure—that supplies the conditions under which inherited dispositions are converted into learning engagement and professional self-belief, whereas regular institutions offer a thinner field. Student origin operationalizes the structural dimension specific to the Chinese context: the persistent urban–rural dual structure generates systematic differences in the composition and saturation of family resources (Tan and Fang, 2023a), so an identical nominal increment of capital is expected to carry a different marginal value across rural and urban families. These two variables therefore capture, respectively, the field-activation condition and the resource-saturation condition that Bourdieu's framework treats as governing capital conversion, which is why they—rather than demographic variables such as gender or grade level—are designated as the focal moderators. On this basis, two directional hypotheses are advanced.

**H4 (directional):** Drawing on the resource-concentration perspective in elite higher education institutions (Whitaker and Wolniak, 2022; Holochwost et al., 2025), the positive effect of cultural capital on professional self-efficacy is hypothesized to be **stronger in key universities than in regular universities**. The theoretical rationale is that key institutions provide field conditions—high-density professional interactions, frequent master-class exposure, and elite peer effects—that activate latent cultural dispositions transmitted by the family into observable professional self-belief, whereas these activation conditions are weaker in regular institutions.

**H5 (directional):** Drawing on the diminishing-marginal-utility framework of family education investment (Jiang, 2024; Wang and Li, 2024), the positive effect of economic capital on learning engagement is hypothesized to be **stronger among rural students than urban students**. The theoretical rationale is that rural families typically operate below the saturation point of music-related expenditure, so each additional unit of economic input produces a larger behavioral incentive, whereas urban families typically operate near the saturation point at which additional input yields smaller behavioral returns.

**Figure 3:**
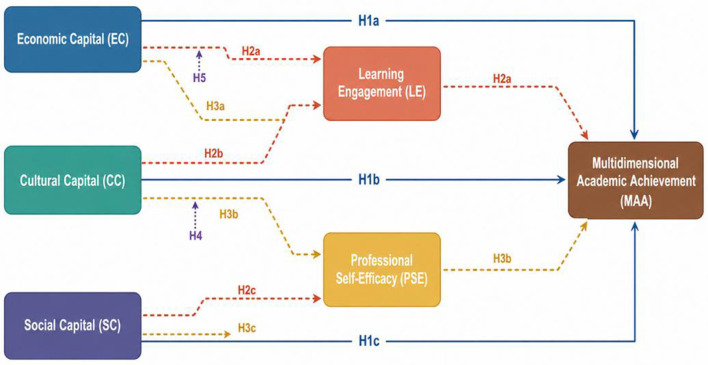
Relationship between research hypotheses and paths.

The corresponding paths of the hypothesized relationships are shown in Figure 3.

### 3.2 Variable operationalization and scale development

The latent variables involved in this study include three types of family capital, learning engagement, professional self-efficacy, and multidimensional academic achievement—six constructs in total. All self-reported items were scored using a 5-point Likert scale, with 1 indicating “completely disagree” and 5 indicating “completely agree.” The development of all scales followed the standardized four-stage procedure recommended by MacKenzie et al. (2011), and is described in detail in Section 3.2.1 below.

The operationalization of family economic capital is designed around the resource-dependence characteristics of the music major. The initial item pool contained four indicators: annual family income, instrument investment, after-school professional training expenditure, and music performance/concert viewing frequency. The operationalization of cultural capital follows Bourdieu's (1986) three-form framework and is adapted to the music education field: embodied cultural capital is measured by parents' music literacy and art appreciation habits, objectified cultural capital by the number of music books and audio-visual materials in the home, and institutionalized cultural capital by parents' educational level. The measurement of social capital covers three dimensions: parent–music teacher contact density, the size of the family's music-related social network, and the breadth of channels through which parents obtain professional development information. The learning engagement scale draws on mature tools in existing educational research and is revised for music majors; the initial pool contained six items covering three sub-dimensions: behavioral engagement (daily practice duration, classroom participation), emotional engagement (enthusiasm for professional learning), and cognitive engagement (use of practice strategies). The professional self-efficacy scale differs from the general academic self-efficacy scale; it specifically measures students' ability beliefs in four aspects: performance/vocal performance, solfeggio, music theory learning, and stage performance.

A key innovation of this study is the multidimensional academic achievement measurement tool. Different from previous studies that used GPA or a single test score, this tool deconstructs the academic achievement of music majors into three independent yet interrelated dimensions: performance skills, theoretical achievement, and artistic practice performance. Importantly, the three achievement dimensions are not based on student self-report. Performance skills are rated independently by two faculty members of each student's main-instrument area using a standardized rubric (see Section 3.2.1); theoretical achievement is obtained directly from official course-grade records of music theory and solfeggio courses; and artistic practice performance is composed of competition awards and faculty evaluation of stage performance over the past academic year. The dimensional weights were determined through expert consultation as described in Section 3.2.1.

A summary of the final operational definitions and measurement indicators of each latent variable (after pilot testing and CFA-based refinement) is shown in Table 1.

**Table 1:** Operationalization and measurement indicators of latent variables (Final Scale).

Latent variable	Dimension	Representative indicators	Items	Source
Economic capital (EC)	Family economic investment	Instrument investment, training expenditure, concert attendance frequency	3	Self-report
Cultural capital (CC)	Embodied/objectified/ institutionalized	Parental musical literacy, music book collection, parental education level	5	Self-report
Social capital (SC)	Social network and information channels	Teacher–parent contact density, music social network, information channels	3	Self-report
Learning engagement (LE)	Behavioral/emotional/cognitive	Practice hours, professional enthusiasm, practice strategy application	5	Self-report
Professional self-efficacy (PSE)	Music professional competence belief	Performance confidence, theory learning confidence, stage confidence	4	Self-report
Multidimensional academic Achievement (MAA)	Performance/theory/practice	Faculty-rated performance score, theory weighted score, artistic practice evaluation	3	Faculty rating and official records

The multidimensional academic achievement scale and the auxiliary self-report scales were jointly developed through a four-stage procedure following MacKenzie et al. (2011).

#### 3.2.1 Stage 1: item generation

An initial item pool of 26 indicators across the six latent variables was generated by reviewing prior music-education achievement frameworks (Wang and Huang, 2024; Jiang and Tong, 2025; Zhang, 2025; Bourdieu, 1986) tripartite cultural capital framework, and the Ministry of Education's curriculum standards for Chinese music undergraduate programs. Items covered three theoretical dimensions of academic achievement: performance skills (initial eight indicators), theoretical knowledge (initial four indicators), and artistic practice (initial four indicators).

#### 3.2.2 Stage 2: expert review (two Delphi rounds)

A panel of seven experts was convened: three music performance professors (each with 15+ years of conservatory teaching experience), two music theory professors, and two educational measurement specialists with prior SEM publications. In Round 1, experts independently rated each item on a 5-point clarity and content-validity scale; items below 4.0 on either dimension were removed. In Round 2, the panel reached consensus on the retained items (Kendall's W = 0.78, *p* < 0.001), and assigned dimension weights for the academic achievement construct using a pairwise comparison procedure: performance skills 0.45, theoretical knowledge 0.30, artistic practice 0.25.

#### 3.2.3 Stage 3: Pilot test (*n* = 85)

The expert-revised version was administered to 85 music undergraduates outside the formal sample between November 2024 and January 2025. Cronbach's α for the six latent variables ranged from 0.81 to 0.88; corrected item–total correlations ranged from 0.48 to 0.79. Two items were flagged for revision based on pilot feedback: the EC item “annual family income” showed a low item–total correlation (*r* = 0.31, likely due to response reluctance for income-related questions), and one reverse-scored LE item showed weak performance (*r* = 0.27). Both items were retained for the formal study to allow further evaluation under a larger sample, but were ultimately removed at the CFA stage (see Section 4.2).

#### 3.2.4 Stage 4: inter-rater reliability for performance skills

Performance-skill scores were obtained from independent faculty ratings rather than student self-report. Each student's main-instrument performance was rated by two faculty members from their institution using a 10-point standardized rubric covering technical accuracy (4 points), musicality and interpretation (4 points), and stage presence (2 points). The two raters worked independently and were blind to each other's scores. Inter-rater reliability was assessed using two-way random-effects intraclass correlation coefficient: ICC (2, *k*) = 0.86, 95% CI [0.83, 0.89], indicating excellent agreement.

### 3.3 Sample selection and data collection

**Figure 4:**
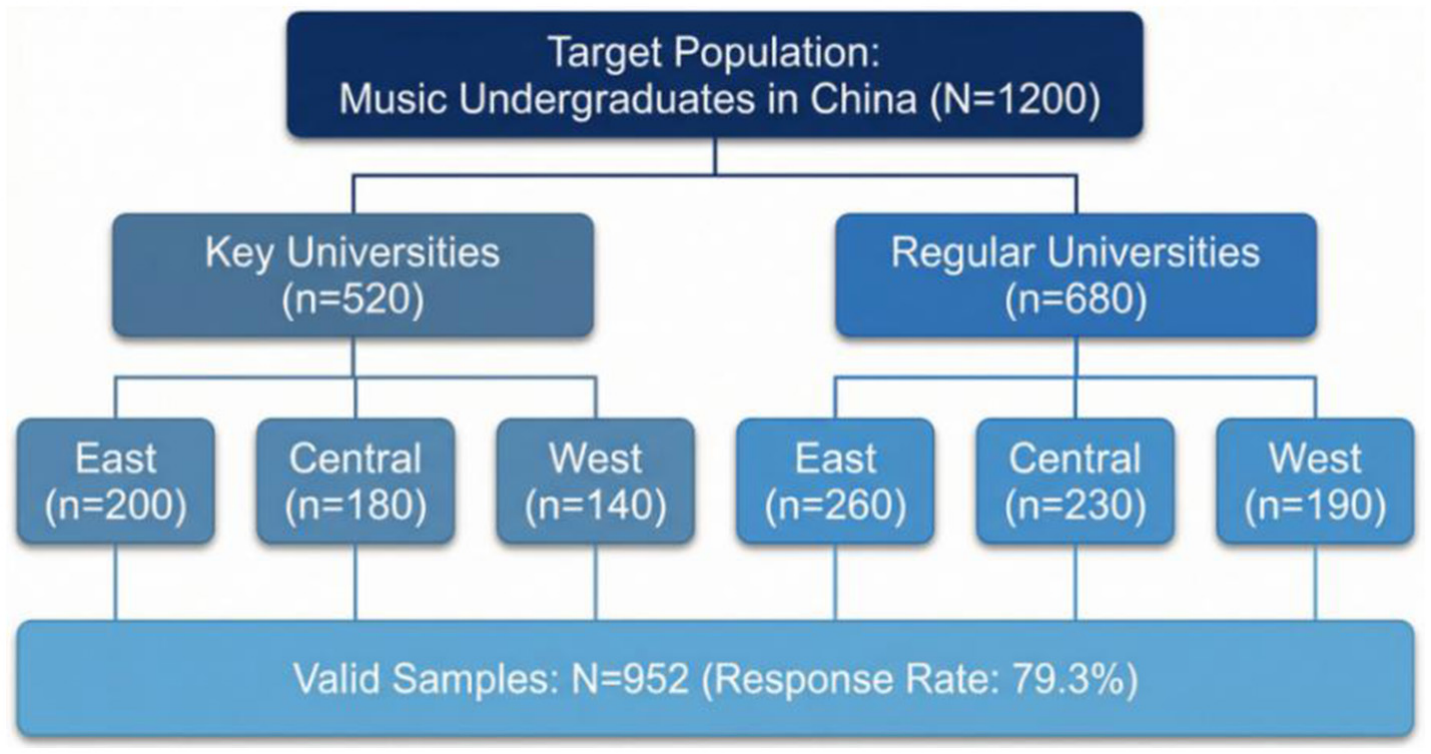
Stratified sampling framework and sample distribution.

The target group of this study is full-time undergraduates majoring in music in Chinese colleges and universities. To ensure the representativeness and heterogeneity of the sample, a strategy combining stratified sampling and convenience sampling was adopted. The stratification basis includes institution type (key institutions and regular institutions) and geographical region (Eastern, Central, and Western China). At each stratum, the music school or art department of the target university was contacted through the researcher's academic cooperation network, and students who voluntarily agreed to participate were recruited to complete the questionnaire. The sampling framework and final sample distribution are shown in Figure 4.

#### 3.3.1 Research timeline

The full research process spanned 26 months, from January 2024 to April 2026:

- Phase 1 (January–April 2024): Literature review and theoretical model construction.
- Phase 2 (May–August 2024): Initial item generation and two-round Delphi expert review.
- Phase 3 (September–October 2024): IRB application and approval. The research protocol was approved by the Institutional Review Board of Geely University of China on 15 October 2024 (Approval No. GUCSPA-REC-2024-0287).
- Phase 4 (November 2024–January 2025): Pilot test with 85 music undergraduates (3 months), scale revision based on pilot results.
- Phase 5 (February 2025): Site permission negotiation with eight cooperating universities; each cooperating institution issued a formal site-access letter.
- Phase 6 (March–September 2025): Main data collection (7 months). Paper questionnaires were administered in the spring semester (March–June 2025); online distribution covered the autumn semester opening period (September 2025).
- Phase 7 (October 2025–January 2026): Data cleaning, CFA, structural model estimation, and multigroup analysis.
- Phase 8 (February–April 2026): Manuscript drafting and submission preparation.

The interval between pilot test completion and the start of main data collection was approximately 2 months, used for instrument finalization and site-access negotiation.

#### 3.3.2 Cooperating universities and sample distribution

Eight universities participated, anonymized as U1–U8 per the data-sharing agreement signed with each institution. The regional and institutional distribution is shown in Table 2.

**Table 2:** Distribution of cooperating universities (*N* = 8).

Region	Key university	Regular university	Subtotal
Eastern	U1 (Shanghai)	U2 (Jiangsu)	2
Central	U3 (Hubei)	U4 (Henan), U5 (Hunan)	3
Western	U6 (Sichuan)	U7 (Shaanxi), U8 (Yunnan)	3
Total	3	5	8

Three universities were classified as key institutions (Project 985/211 or “Double First-Class” status) and five as regular institutions, following the Ministry of Education tiering system. The three key institutions contributed 426 valid responses (44.7% of *N* = 952), and the five regular institutions contributed 526 valid responses (55.3%).

#### 3.3.3 Sample size considerations

With six latent constructs and 23 final observed indicators, the target effective sample size was set to a minimum of 800 to ensure adequate power for multigroup invariance testing across two grouping variables (no less than 200 per group; Cheah et al., 2023; Hair et al., 2022). This study planned to distribute 1,200 questionnaires across the 8 cooperating universities, targeting an effective response rate of at least 80%.

**Figure 5:**
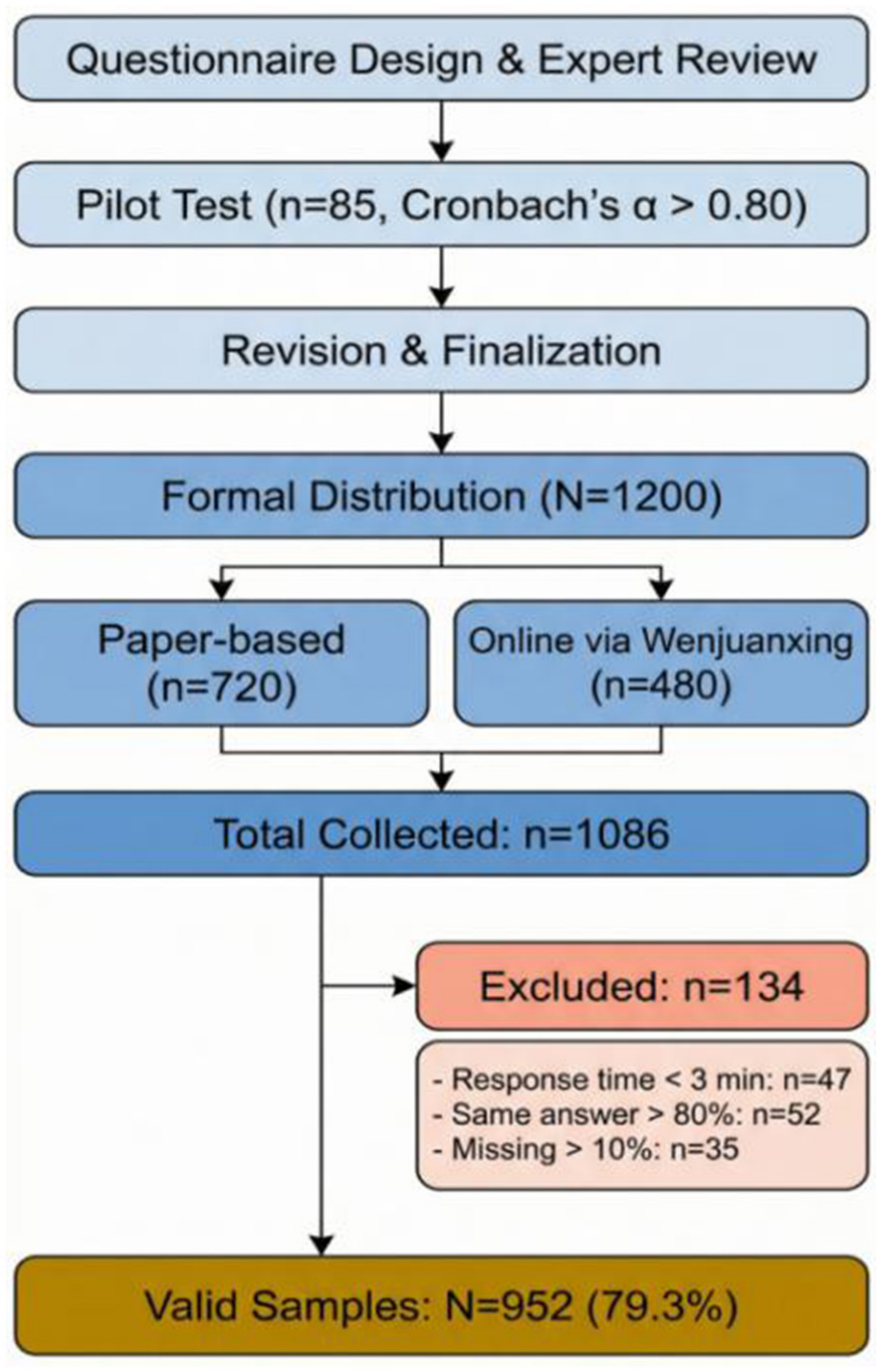
Flow chart of data collection and screening.

Data collection was conducted in parallel through offline paper questionnaires and online electronic questionnaires. The paper questionnaire was distributed centrally in specialized course classrooms of the cooperating universities and completed under the guidance of trained research assistants (who were graduate students from the local institution, ensuring on-site oversight). The online questionnaire was generated via the Wenjuanxing platform, with the QR-coded link posted on official bulletin boards of the music schools. All participants—who were adult undergraduate students aged 18 or above—read and signed the informed consent form themselves before participation. No guardian consent was sought or required, as all participants were legally adults capable of providing autonomous informed consent. After data collection, invalid responses with completion time below 3 minutes, single-option selection rate above 80%, or missing values above 10% were excluded. The data collection and filtering process is shown in Figure 5.

#### 3.3.4 Ethical approval across multiple sites

Although the principal IRB approval was issued by the Institutional Review Board of Geely University of China (Approval No. GUCSPA-REC-2024-0287), site-access and institutional consent letters were independently obtained from each of the seven other cooperating universities prior to data collection. None of the seven institutions required separate ethical review; this was confirmed in writing by each institution's research ethics office, citing the single-IRB review principle for multi-site, minimal-risk educational research. All on-site data collection was conducted by trained research assistants who were graduate students or faculty staff of the respective cooperating universities, ensuring local oversight throughout the data collection period. The research protocol, informed consent procedures, and data confidentiality protections complied with the Declaration of Helsinki and the Ethical Review Measures for Life Sciences and Medical Research Involving Human Subjects (China, 2023).

### 3.4 Structural equation modeling analysis strategy

The structural equation model was analyzed in two steps: the first step was to test the measurement model, and the second step was to test the structural model. All analyses were performed using AMOS 28.0 with maximum likelihood (ML) estimation.

#### 3.4.1 Measurement model verification

**Figure 6:**
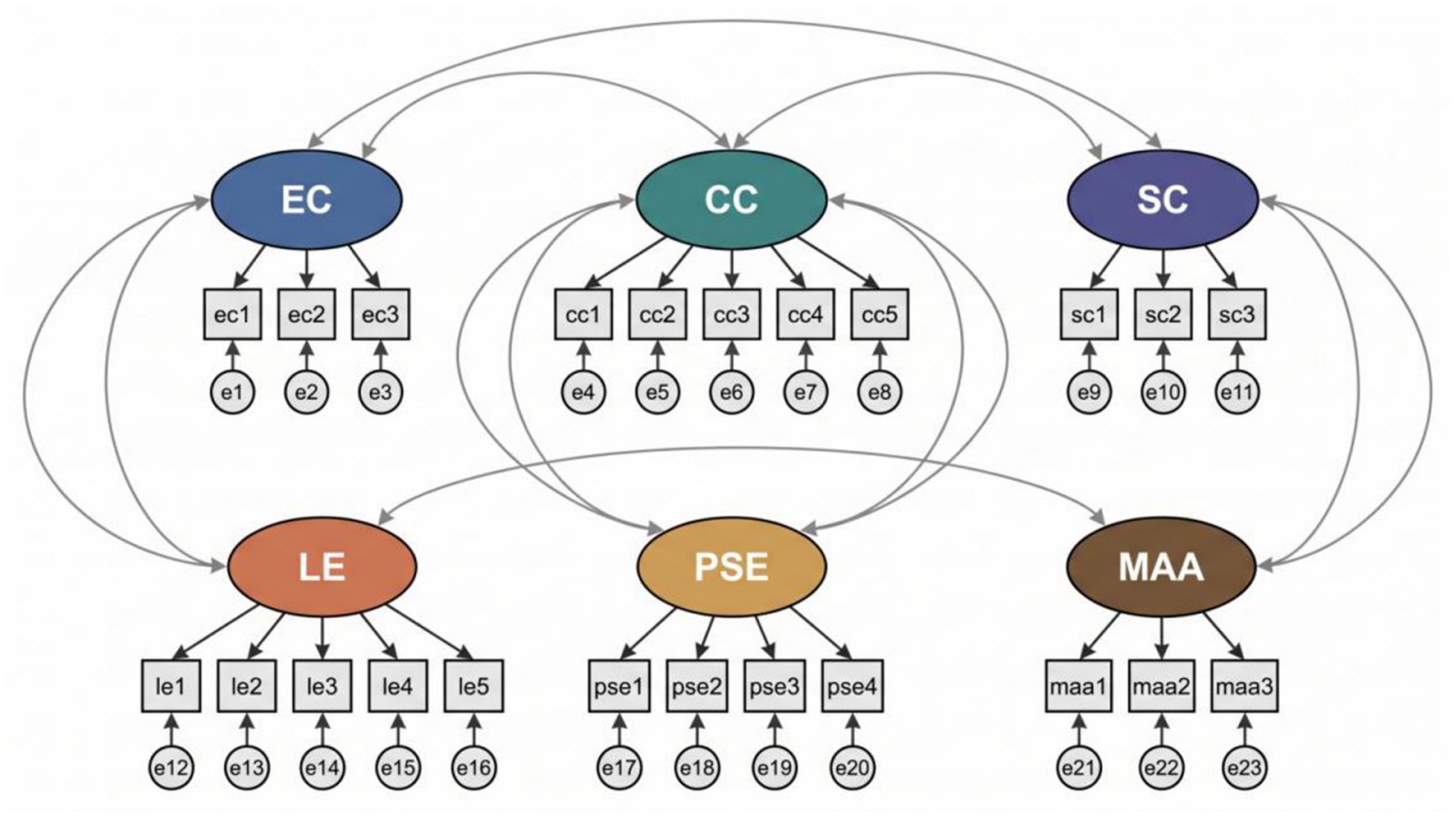
Structure of the confirmatory factor analysis measurement model.

Confirmatory factor analysis (CFA) was performed on the six latent variables to evaluate factor loadings, composite reliability (CR), and average variance extracted (AVE) of each construct. Items with factor loadings below 0.5 were deleted. CR values should exceed 0.7, and AVE values should exceed 0.5 (Zheng and Bentler, 2025). Overall model fit was comprehensively evaluated by the following indices: χ^2^*/df* (should be below 3), CFI (should exceed 0.90), TLI (should exceed 0.90), RMSEA (should be below 0.08), and SRMR (should be below 0.08). The structure of the CFA measurement model is shown in Figure 6.

#### 3.4.2 Structural model verification

On the basis of good measurement model fit, a complete structural model containing both direct and mediation effects was constructed. In the structural model, the path coefficient of the direct effect of three-dimensional family capital on academic achievement is expressed as γ_*ij*_, where *i* indexes the *i*-th exogenous latent variable (*i* = 1, 2, 3 corresponding to EC, CC, SC respectively), and *j* indexes the endogenous latent variable MAA. The bootstrap method was used to test mediation effects, with 5,000 resamples and a 95% bias-corrected percentile confidence interval. If the confidence interval does not contain zero, the mediation effect is significant. The indirect effect is calculated as (Equation 1):


$$\begin{array}{l} IE_{im}=a_{im}b_{m} \end{array}$$ (1)

where *IE*_*im*_ represents the indirect effect of the *i*-th exogenous latent variable on MAA transmitted through the mediator *m* (*m* = LE or PSE); *a*_*im*_ is the path coefficient from the *i*-th exogenous latent variable to mediator *m*; *b*_*m*_ is the path coefficient from mediator *m* to MAA. The total effect is the sum of direct and indirect effects (Equation 2):


$$\begin{array}{l} TE_{i}=\gamma_{ij}+\overset{2}{\underset{m=1}{\sum }}a_{im}b_{m} \end{array}$$ (2)

where *TE*_*i*_ is the total effect of the *i*-th exogenous latent variable on MAA, γ_*ij*_ is the direct effect coefficient, and the summation term aggregates the indirect effects through the two mediators.

#### 3.4.3 Multigroup comparative analysis

**Figure 7:**
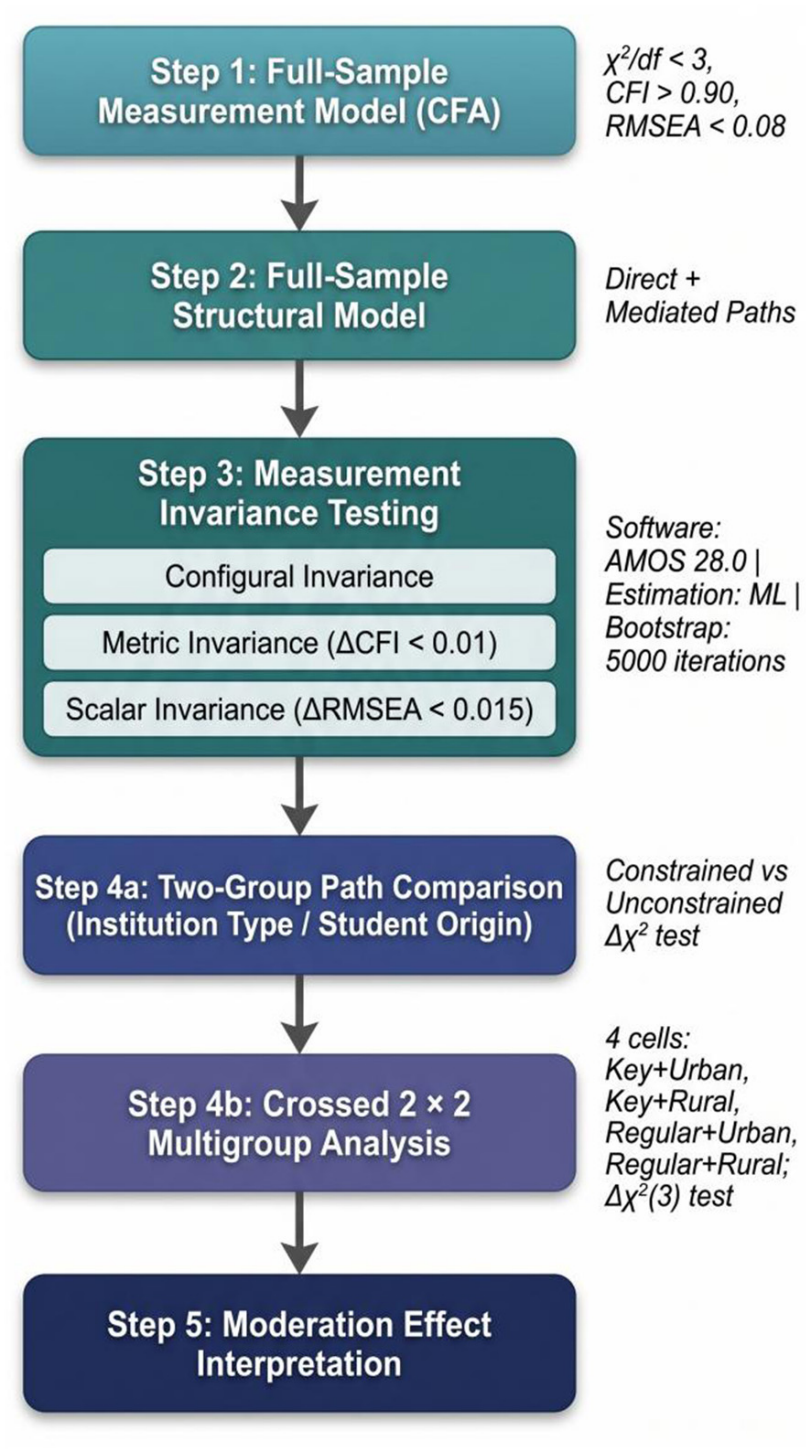
Multigroup structural equation model analysis flow chart.

One of the key innovations of this study is the use of the multigroup structural equation model to test the moderating effects of institution type and student origin. Before comparing path coefficients between groups, measurement invariance must be tested step by step (Perez Alonso et al., 2024). The invariance test proceeds through three progres levels: configural invariance (each group has the same factor structure), metric invariance (each group has the same factor loadings), and scalar invariance (each group has the same loadings and intercepts). The decision criterion is: when |Δ CFI| < 0.01 and |Δ RMSEA| < 0.015 between nested models, the more stringent invariance level is supported. Once measurement invariance holds, chi-square difference tests are performed by constraining and releasing path coefficients to identify paths with significant between-group differences. The test statistic for path coefficient differences is (Equation 3):


$$\begin{array}{l} \text{Δ}\chi^{2}=\chi_{t}^{2}-\chi_{u}^{2} \end{array}$$ (3)

where $\chi_{t}^{2}$ is the chi-square value of the constrained model (the path being tested is constrained equal across groups) and $\chi_{u}^{2}$ is the chi-square value of the unconstrained model. This statistic follows a chi-square distribution with degrees of freedom equal to the difference in degrees of freedom. If Δχ^2^ is significant at Δ*df(p* < 0.05), the path coefficient differs between groups. The stepwise verification procedure for multigroup analysis is shown in Figure 7.

#### 3.4.4 Common method bias control

Common method bias (CMB) was addressed at both the design stage and the analysis stage. At the design stage, the three dependent variables in the multidimensional academic achievement construct were measured using non-self-report sources (Section 3.2.1): performance skills came from independent faculty rating (ICC = 0.86), theoretical achievement from official course-grade records, and artistic practice from faculty evaluation combined with award records. This multi-source measurement design substantially reduces CMB risk by separating the dependent variables from the self-reported independent and mediator variables. Procedural controls for the remaining self-reported scales (EC, CC, SC, LE, PSE) included anonymous administration, reverse-scored items, and attention-check items placed between different constructs.

At the analysis stage, three complementary statistical procedures were applied to evaluate residual CMB risk on the self-reported variables:

(1) **Harman's single-factor test** as a preliminary screen (Fuller et al., 2016).
(2) **The Unmeasured Latent Method Construct (ULMC) approach (Williams et al., 2010)**: an additional latent method factor was added to the CFA, with all observed indicators loading on both their theoretical construct and the method factor. The change in model fit and substantive path coefficients before and after adding the method factor was examined.
(3) **The marker variable test (Lindell and Whitney**, **2001****)**: a theoretically unrelated single-item indicator (preference for outdoor sports) was included in the questionnaire as a marker. The partial correlations between the marker and substantive variables were examined; near-zero partial correlations would indicate negligible CMB contamination.

#### 3.4.5 Endogeneity and robustness checks

Because the present design is cross-sectional, the possibility of reverse causality—that high-achieving students attract greater family investment, or that professional self-efficacy is a product rather than a cause of prior attainment—was addressed at three levels rather than left unexamined. First, the three exogenous constructs are deliberately operationalized through pre-determined, low-reversibility indicators (parental education level, parental musical literacy, instrument ownership, and home cultural resources; Section 3.2) that are temporally prior to and largely fixed relative to a student's current university achievement, so the direction running from family capital to achievement is the substantively more defensible one. Second, the dependent variable is measured entirely from non-self-report sources—independent faculty ratings, official course-grade records, and award records (Section 3.2.1)—which severs the same-source loop through which current achievement could mechanically inflate self-reported capital or self-efficacy. Third, because the analytic sample consists exclusively of students already admitted through China's unified music entrance examination, raw economic capital is subject to upstream range restriction within the enrolled population, which is itself one reason the residual economic-capital effect is modest; to confirm that the structural estimates are not driven by observed compositional differences, the full structural model was re-estimated with grade level, gender, region, and institution type entered as covariates on both mediators and the outcome, and a reverse-direction specification (academic achievement predicting family capital) was estimated for comparison, with results reported in Section 4.3. These procedures mitigate, though they cannot fully eliminate, endogeneity; a longitudinal or instrumental-variable design is recommended for confirmatory work, as noted in Section 6.

**Figure 8:**
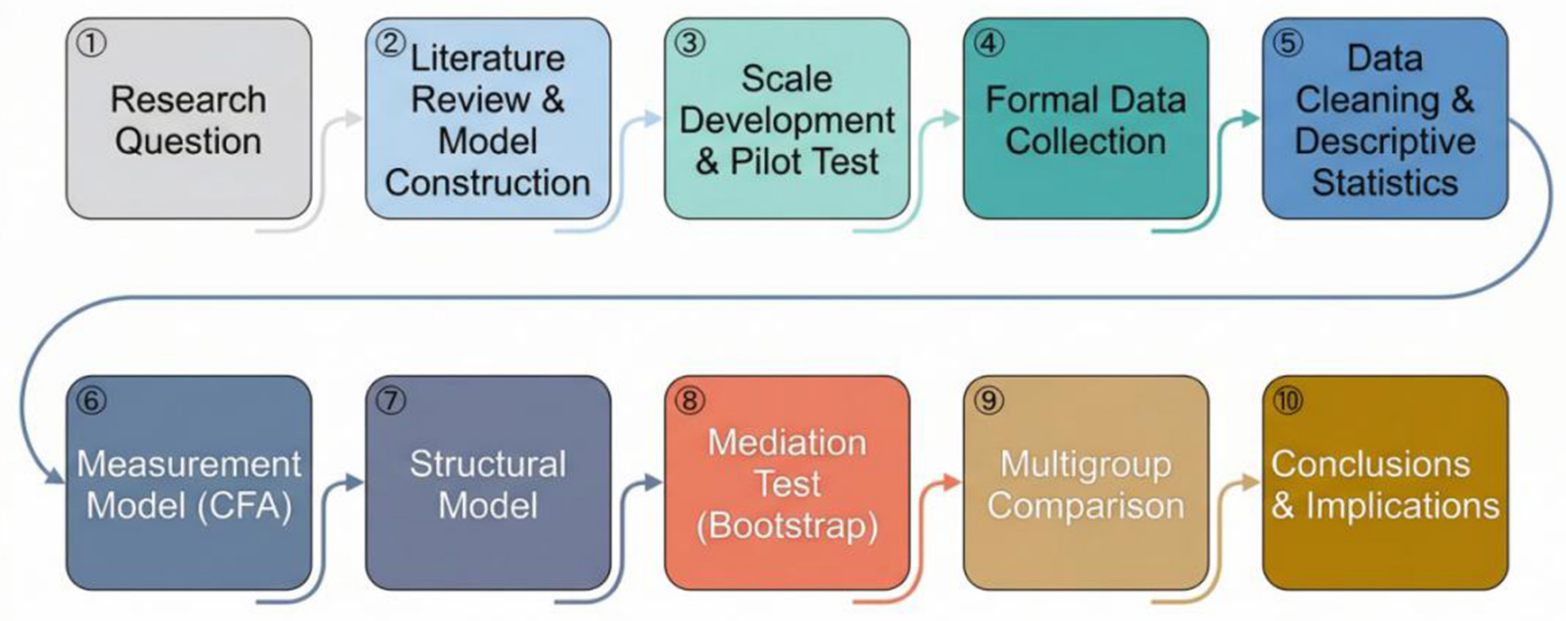
Overall technical roadmap of the study.

The overall technical roadmap of the study is shown in Figure 8, and the goodness-of-fit criteria for the structural equation model are summarized in Table 3.

**Table 3:** Goodness-of-fit criteria for structural equation models.

Fit index	Acceptable criterion	Good criterion	Interpretation
χ^2^/*df*	< 5	< 3	Overall chi-square fit test
CFI	>0.85	>0.90	Comparative Fit Index
TLI	>0.85	>0.90	Tucker–Lewis Index
RMSEA	< 0.10	< 0.08	Root mean square error of approximation
SRMR	< 0.10	< 0.08	Standardized root mean square residual

## 4 Research results

### 4.1 Descriptive statistics and sample characteristics

A total of 1,200 questionnaires were distributed and 1,086 were returned. After eliminating invalid responses, 952 valid samples were obtained, yielding an effective response rate of 79.3%. Of the sample, women accounted for 61.8% (*n* = 588) and men 38.2% (*n* = 364); freshmen to seniors accounted for 22.1%, 27.4%, 28.6% and 21.9% respectively; students from key universities accounted for 44.7% (*n* = 426) and students from regular universities 55.3% (*n* = 526); urban students accounted for 57.5% (*n* = 547) and rural students 42.5% (*n* = 405). The cross-tabulation of institution type and student origin yields four subgroups that will be used in the moderated mediation analysis (Section 4.5), with sample sizes as shown in Table 4.

**Table 4:** Cross-tabulation of institution type and student origin (*N* = 952).

Crossed subgroup	*n*	Percentage
Key university + urban	281	29.5%
Key university + rural	145	15.2%
Regular university + urban	266	27.9%
Regular university + rural	260	27.3%
Total	952	100.0%

All four subgroups exceed the minimum cell size of 100 recommended for crossed multigroup SEM (Hair et al., 2022). The descriptive statistical results of each latent variable are shown in Table 5.

**Table 5:** Descriptive statistics of latent variables (*N* = 952).

Variable	Mean	SD	Skewness	Kurtosis	Min	Max
Economic capital (EC)	2.83	0.91	0.14	−0.42	1	5
Cultural capital (CC)	3.21	0.86	−0.18	−0.29	1	5
Social capital (SC)	2.76	0.94	0.22	−0.51	1	5
Learning engagement (LE)	3.47	0.78	−0.31	−0.08	1.17	5
Professional self-efficacy (PSE)	3.12	0.83	−0.09	−0.36	1	5
Multidimensional academic achievement (MAA)	3.38	0.74	−0.22	−0.15	1.33	5

**Figure 9:**
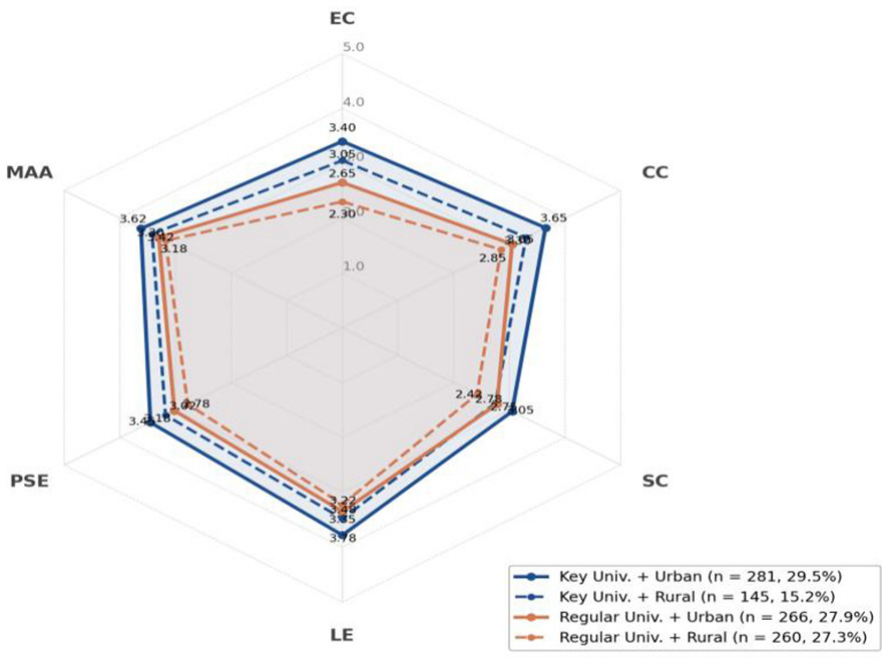
Radar chart comparing the mean values of latent variables across the four crossed subgroups (key university + urban, *n* = 281; key university + rural, *n* = 145; regular university + urban, *n* = 266; regular university + rural, *n* = 260).

As Table 5 shows, the absolute values of skewness and kurtosis of all variables are less than 2 and 7 respectively, meeting the basic requirements for the normality assumption. Among the three types of family capital, cultural capital has the highest mean (*M* = 3.21), social capital the lowest (*M* = 2.76), and economic capital lies in between (*M* = 2.83). This distribution suggests that music majors' families have relatively rich cultural-resource accumulation, while social-network resource allocation shows greater individual variation. The mean distribution of latent variables across the four crossed subgroups is shown in Figure 9.

### 4.2 Measurement model testing

Confirmatory factor analysis was performed on the six latent variables. In the initial model, the factor loadings of two items fell below the critical value of 0.5: the “annual family income” item in the EC scale (λ = 0.46) and the reverse-scored item in the LE scale (λ = 0.43). After their removal, the model was re-estimated. The fit of the revised measurement model is good: χ^2^*/df* = 2.37, CFI = 0.94, TLI = 0.93, RMSEA = 0.051, SRMR = 0.042. The item-level standardized factor loadings (λ), communalities (*h*^2^ = λ^2^), and construct-level reliability and convergent validity indices for the final 23-indicator model are jointly reported in Table 6.

**Table 6:** Reliability and validity of the measurement model—item-level loadings, communalities, and construct-level indices (*N* = 952).

Construct	Item	Item description (abridged)	λ	h^2^	α	CR	AVE
Economic capital (EC)	ec1	Instrument investment	0.84	0.706	0.81	0.82	0.60
	ec2	Professional training expenditure	0.76	0.578			
	ec3	Concert/performance viewing frequency	0.68	0.462			
Cultural capital (CC)	cc1	Parental music literacy (embodied)	0.85	0.723	0.86	0.87	0.58
	cc2	Art appreciation habits at home (embodied)	0.83	0.689			
	cc3	Music books at home (objectified)	0.78	0.608			
	cc4	Audio-visual material collection (objectified)	0.72	0.518			
	cc5	Parental education level (institutionalized)	0.62	0.384			
Social capital (SC)	sc1	Parent–music teacher contact density	0.88	0.774	0.83	0.84	0.64
	sc2	Family music-related social network size	0.80	0.640			
	sc3	Breadth of professional information channels	0.71	0.504			
Learning engagement (LE)	le1	Average daily practice duration	0.82	0.672	0.85	0.86	0.55
	le2	Professional learning enthusiasm	0.80	0.640			
	le3	Classroom participation	0.74	0.548			
	le4	Strategic practice planning	0.69	0.476			
	le5	Cognitive practice strategy use	0.64	0.410			
Professional self-efficacy (PSE)	pse1	Performance/vocal performance confidence	0.87	0.757	0.88	0.89	0.67
	pse2	Solfeggio confidence	0.86	0.740			
	pse3	Music theory learning confidence	0.80	0.640			
	pse4	Stage performance confidence	0.74	0.548			
MAA	maa1	Performance skills (faculty-rated, 10-pt rubric)	0.81	0.656	0.79	0.80	0.57
	maa2	Theoretical achievement (course-grade records)	0.76	0.578			
	maa3	Artistic practice (awards + faculty evaluation)	0.69	0.476			

All standardized factor loadings are significant at *p* < 0.001. Communality (*h*^2^) is computed as the square of the standardized loading. The complete item wording is provided in Appendix A.

**Figure 10:**
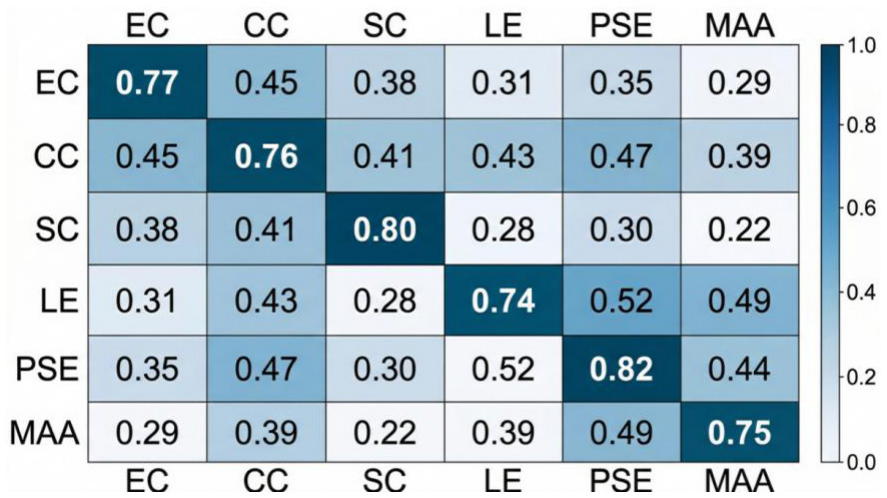
Heatmap of correlation coefficients between latent variables with the square root of AVE annotated on the diagonal.

As Table 6 shows, all 23 item-level standardized loadings range from 0.62 to 0.88, well above the 0.50 threshold. Communalities range from 0.384 to 0.774, indicating that each retained indicator captures a substantial proportion of its construct's variance. Cronbach's α values for all latent variables exceed 0.79 and CR values exceed 0.80, indicating good internal consistency and composite reliability. All AVE values exceed 0.55, indicating that the latent variables explain their observed indicators above the acceptable level. For discriminant validity, the square root of each latent variable's AVE exceeds the correlation coefficients between this variable and other variables, satisfying the Fornell–Larcker criterion. The comparison between the inter-construct correlation matrix and the square root of AVE is shown in Figure 10.

#### 4.2.1 Common method bias diagnostics

The results of Harman's single-factor test showed that the variance proportion explained by the first factor in the unrotated exploratory factor analysis was 28.6%, well below the 40% critical value, providing initial evidence that CMB is not severe.

To further verify this conclusion, the Unmeasured Latent Method Construct (ULMC) test (Williams et al., 2010) was conducted on the five self-reported latent variables (EC, CC, SC, LE, PSE). When an additional latent method factor was added with all self-reported indicators loading on both their theoretical construct and the method factor, the improvement in model fit was minimal (Δ CFI = 0.006, Δ RMSEA = 0.002), and the substantive standardized path coefficients changed by less than 0.02 in absolute magnitude. The marker-variable test (Lindell and Whitney, 2001) using preference for outdoor sports as the marker showed partial correlations with substantive variables ranging from |r| = 0.03 to 0.08, none reaching statistical significance. Together, the three diagnostic procedures confirm that CMB does not pose a substantive threat to the validity of the findings, a conclusion further reinforced by the multi-source measurement design of the dependent variable (Section 3.2.1).

#### 4.2.2 Mode-effect test for paper vs. online administration

To address the potential concern that paper-based and online questionnaire administration might yield non-equivalent responses, a two-group measurement invariance test was conducted across the two administration modes (paper *n* = 572 valid; online *n* = 380 valid). Configural invariance (χ^2^/df = 2.41, CFI = 0.937, RMSEA = 0.052), metric invariance (Δ CFI = −0.004, Δ RMSEA = 0.001), and scalar invariance (Δ CFI = −0.006, Δ RMSEA = 0.002) were all supported, indicating that respondents across the two modes interpreted and responded to the items in a measurement-equivalent manner. Latent mean comparisons of the five self-reported variables between modes yielded no significant differences (all *p* > 0.10). The mode of administration therefore did not bias the substantive findings.

### 4.3 Structural model and path analysis

Based on the verification of the measurement model, a complete structural equation model was constructed. The overall fit indices of the structural model are: χ^2^/df = 2.51, CFI = 0.93, TLI = 0.92, RMSEA = 0.054, SRMR = 0.048. All indices meet the good-fit standard. The standardized coefficients and significance tests for each path are shown in Table 7.

**Table 7:** Standardized path coefficients of the structural model.

Path	β	SE	C.R.	*p*	Hypothesis	Result
EC → MAA	0.12	0.04	2.86	0.004	H1a	Supported
CC → MAA	0.19	0.05	3.92	< 0.001	H1b	Supported
SC → MAA	0.08	0.04	1.88	0.060	H1c	Not supported
EC → LE	0.15	0.04	3.41	< 0.001	—	—
CC → LE	0.28	0.05	5.74	< 0.001	—	—
SC → LE	0.11	0.04	2.52	0.012	—	—
EC → PSE	0.18	0.04	4.12	< 0.001	—	—
CC → PSE	0.31	0.05	6.23	< 0.001	—	—
SC → PSE	0.14	0.04	3.18	0.001	—	—
LE → MAA	0.34	0.05	7.16	< 0.001	—	—
PSE → MAA	0.27	0.05	5.52	< 0.001	—	—

**Figure 11:**
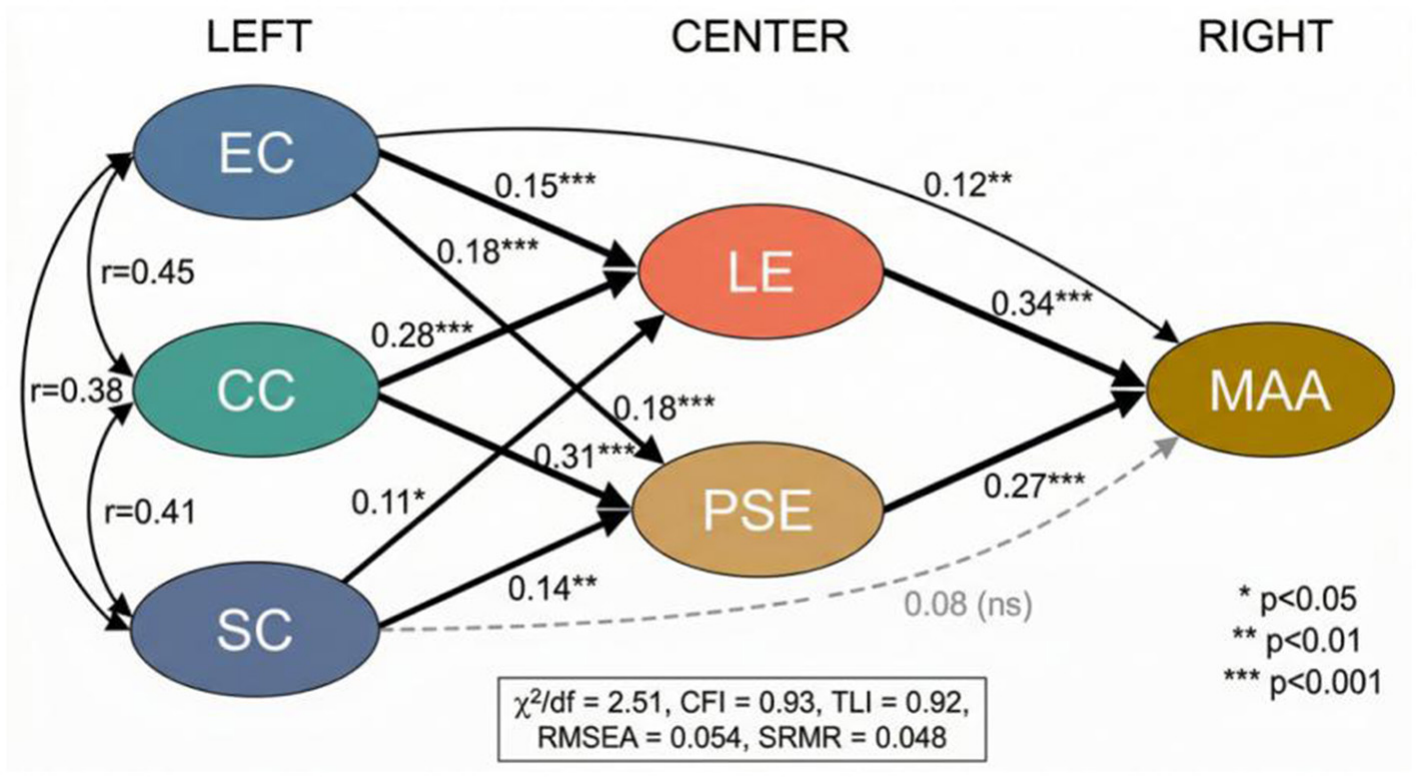
Standardized path coefficients of the structural equation model.

Table 7 reveals several important findings. In terms of direct effects, cultural capital exerts the strongest direct effect on multidimensional academic achievement (β = 0.19, *p* < 0.001), followed by economic capital (β = 0.12, *p* = 0.004), while the direct effect of social capital does not reach statistical significance (β = 0.08, *p* = 0.060). It should be noted that the SC → MAA coefficient reported here is estimated within the full structural model with both mediators (LE and PSE) included simultaneously. This is the correct specification for evaluating the residual direct effect of SC after accounting for the mediation pathways, and it is on the basis of this specification—rather than a bivariate test—that the mediation type for the SC → MAA relationship will be classified in Section 4.4 (Hayes, 2018; Zhao et al., 2010). This finding indicates that family cultural capital is the core family-level factor predicting music majors' academic performance, while the influence of social capital may be realized indirectly through behavioral and psychological mediators. In terms of effects on the mediators, the path coefficients from cultural capital to learning engagement (β = 0.28) and to professional self-efficacy (β = 0.31) are the highest, indicating that parents' music literacy and the family cultural atmosphere play a leading role in stimulating students' learning motivation and shaping their professional confidence. The complete standardized path coefficients are shown in Figure 11.

To assess the possibility of endogeneity raised in Section 3.4, two robustness analyses were conducted. First, the full structural model was re-estimated with student grade level, gender, region, and institution type entered as covariates on both mediators and the outcome. The overall fit remained acceptable (χ^2^/df = 2.46, CFI = 0.926, TLI = 0.917, RMSEA = 0.053, SRMR = 0.049), and every focal coefficient retained its sign, significance, and rank order, with changes in standardized estimates not exceeding 0.01 in absolute value: the CC → MAA path moved from 0.19 to 0.18 (*p* < 0.001), EC → MAA from 0.12 to 0.13 (*p* = 0.003), and SC → MAA remained non-significant (0.08 to 0.07, *p* = 0.071), while the capital-to-mediator and mediator-to-outcome paths were similarly stable. The inversion between cultural and economic capital therefore persists after adjustment, indicating that the substantive conclusions are not attributable to observed compositional differences across grades, genders, regions, or institution types. Second, a reverse-direction model specifying multidimensional academic achievement as a predictor of the three family-capital constructs was estimated for comparison; it produced a clearly inferior fit (χ^2^/df = 3.47, CFI = 0.879, TLI = 0.866, RMSEA = 0.073, SRMR = 0.070), with the comparative fit indices falling below conventional thresholds, providing additional, if not conclusive, support for the assumed ordering from family capital to achievement. Taken together with the pre-determined nature of the capital indicators and the non-self-report measurement of the dependent variable (Section 3.2.1), these checks indicate that reverse causality is unlikely to account for the observed pattern of effects.

### 4.4 Mediation effect tests

The bootstrap method (5,000 resamples) was used to test the mediation effects of learning engagement and professional self-efficacy. The 95% bias-corrected percentile confidence intervals are shown in Table 8.

**Table 8:** Bootstrap test results for mediation effects.

Mediation path	Indirect effect	Boot SE	95% CI lower	95% CI upper	Hypothesis	Result
EC → LE → MAA	0.051	0.018	0.020	0.089	H2a	Supported
CC → LE → MAA	0.095	0.024	0.052	0.145	H2b	Supported
SC → LE → MAA	0.037	0.017	0.008	0.074	H2c	Supported
EC → PSE → MAA	0.049	0.016	0.021	0.083	H3a	Supported
CC → PSE → MAA	0.084	0.022	0.045	0.130	H3b	Supported
SC → PSE → MAA	0.038	0.015	0.012	0.070	H3c	Supported

Table 8 shows that the 95% confidence intervals of all six mediation paths exclude zero, confirming that all six mediation effects are statistically significant. Within the LE pathway, the indirect effect of cultural capital on academic achievement is the largest (0.095), followed by economic capital (0.051) and social capital (0.037). Within the PSE pathway, the order is cultural capital (0.084) >economic capital (0.049) >social capital (0.038).

To facilitate evaluation of the relative importance of each mediation pathway, Table 9 reports the effect-size decomposition by direct effect, total indirect effect, total effect, and the variance accounted for (VAF) ratio (indirect/total), following Hair et al. (2017) and Zhao et al. (2010).

**Table 9:** Effect decomposition and mediation classification.

Capital	Direct effect	Indirect via LE	Indirect via PSE	Total effect	VAF (%)	Mediation type
EC → MAA	0.120	0.051	0.049	0.220	45.5%	Partial (complementary)
CC → MAA	0.190	0.095	0.084	0.369	48.5%	Partial (complementary)
SC → MAA	0.080 (n.s.)	0.037	0.038	0.155	100.0%	Full (indirect-only)

Following the classification scheme of Zhao et al. (2010) and Hair et al. (2017): VAF below 20% indicates no mediation; 20%−80% indicates partial mediation; and a non-significant direct path combined with significant indirect path(s) indicates indirect-only (full) mediation. According to these criteria, both EC and CC exhibit complementary partial mediation (both direct and indirect paths significant and in the same direction), while SC exhibits indirect-only full mediation (direct path non-significant within the full structural model, both indirect paths significant). This classification is statistically rigorous: the judgment of full mediation for SC is not based on a bivariate test but on the direct path coefficient estimated within the full model that already includes the mediators, consistent with Hayes's (2018) definition of full mediation.

**Figure 12:**
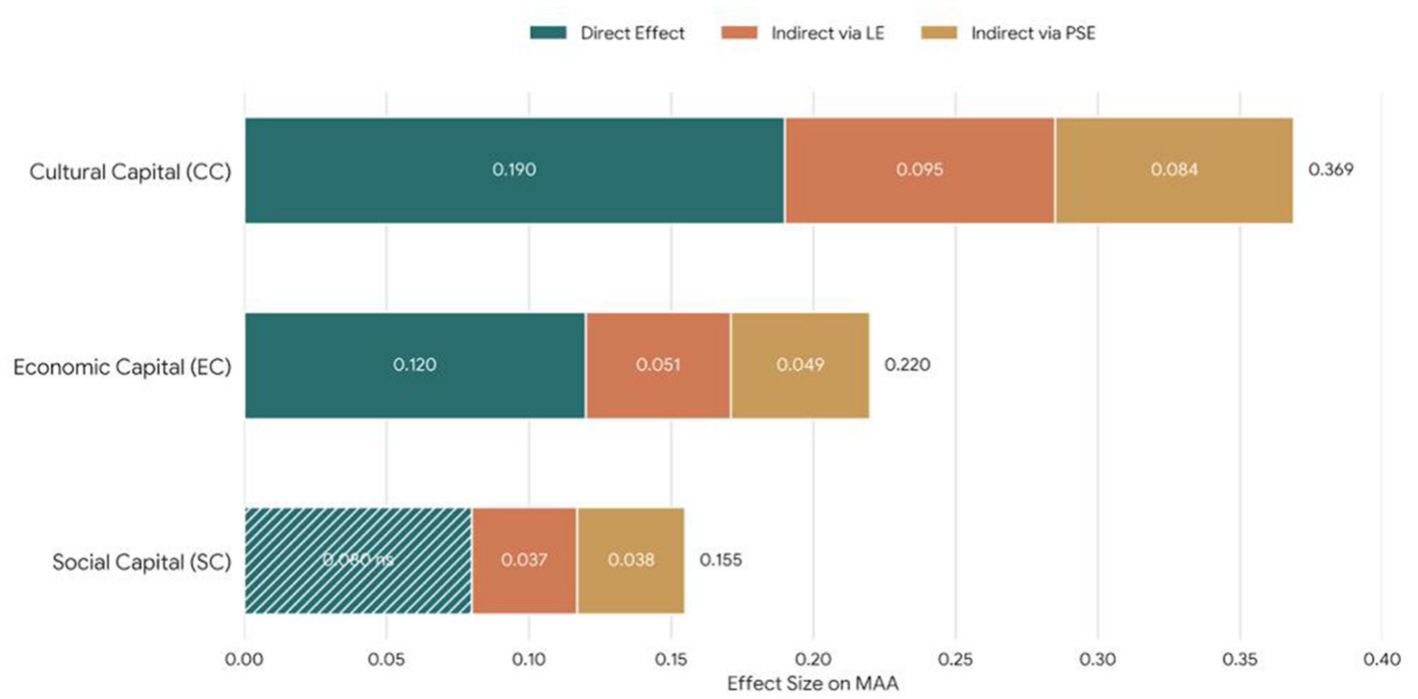
Decomposition and stacked bar chart of direct, indirect, and total effects across the three types of family capital. Hatched pattern indicates ns direct effect. SC → MAA exhibits indirect-only (full) mediation per Zhao et al. (2010): VAF = 100%. EC and CC exhibit complementary partial mediation (VAF = 45.5 and 48.5%, respectively).

The decomposition of direct, indirect, and total effects across the three types of family capital is shown in Figure 12.

### 4.5 Multigroup comparative analysis

#### 4.5.1 Measurement invariance tests

Multigroup measurement invariance was tested step by step by institution type (key vs. regular) and by student origin (urban vs. rural). As shown in Table 10, configural, metric, and scalar invariance are all supported under the two grouping schemes (|Δ CFI| < 0.01 and |Δ RMSEA| < 0.015), satisfying the pre-conditions for between-group path coefficient comparison.

**Table 10:** Measurement invariance test results.

Grouping	Invariance level	χ^2^	*df*	CFI	ΔCFI	RMSEA	ΔRMSEA
Institution type	Configural	648.3	362	0.932	—	0.058	—
	Metric	671.5	379	0.929	−0.003	0.057	−0.001
	Scalar	702.8	396	0.924	−0.005	0.058	0.001
Student origin	Configural	641.7	362	0.934	—	0.057	—
	Metric	663.2	379	0.931	−0.003	0.056	−0.001
	Scalar	691.6	396	0.926	−0.005	0.057	0.001

#### 4.5.2 Two-group path coefficient differences

**Figure 13:**
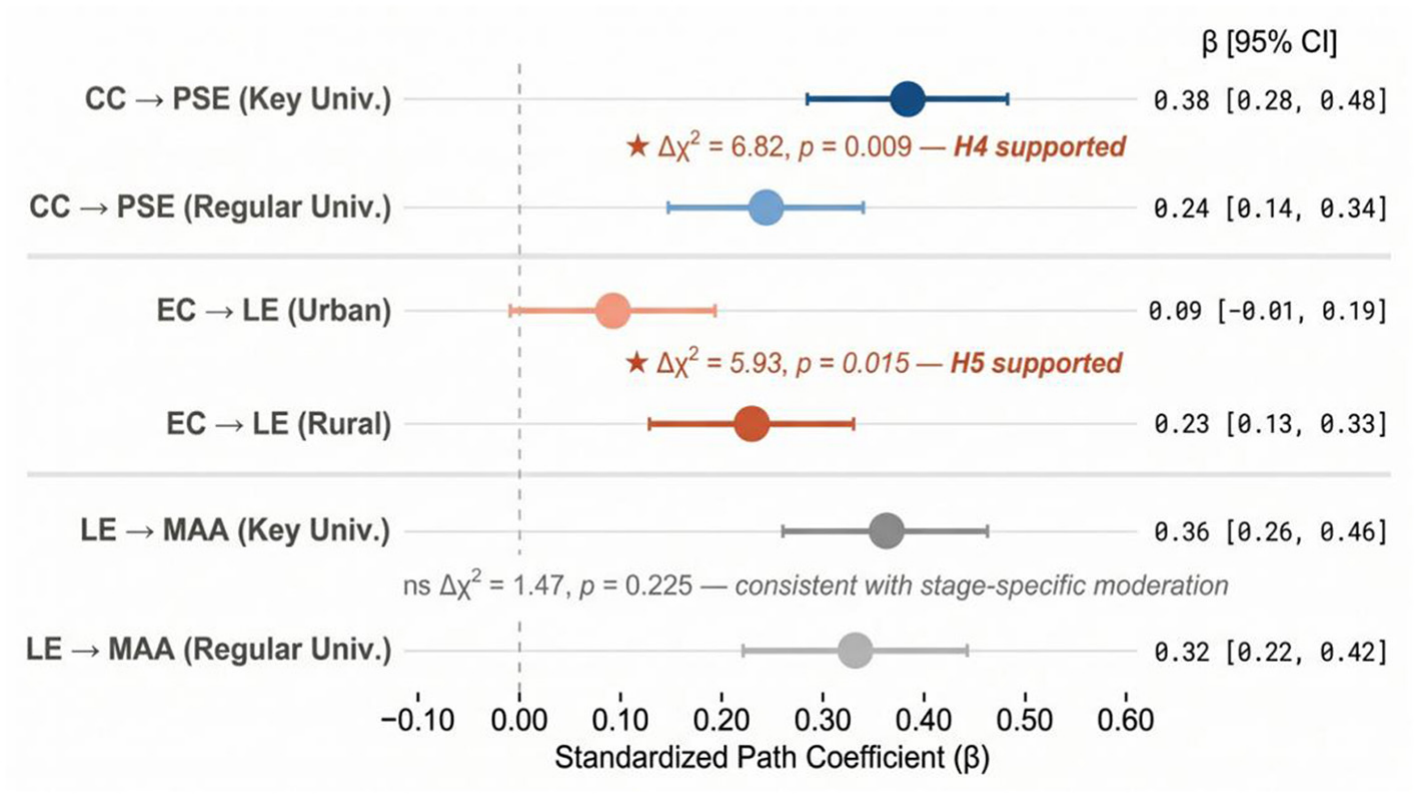
Forest plot comparing the key path coefficient differences between institution-type and student-origin groupings.

Once measurement invariance held, chi-square difference tests were performed by constraining each path coefficient one at a time to identify paths with significant between-group differences. The results show that in the institution-type grouping, the path coefficient of cultural capital on professional self-efficacy in the key university group (β = 0.38) is significantly higher than that in the regular university group (β = 0.24), Δχ^2^ = 6.82, Δ*df* = 1, *p* = 0.009, supporting H4 in the hypothesized direction. There was no significant difference in the LE → MAA path between the two groups (Δχ^2^ = 1.47, *p* = 0.225). In the student-origin grouping, the path coefficient of economic capital on learning engagement in the rural group (β = 0.23) is significantly higher than that in the urban group (β = 0.09), Δχ^2^ = 5.93, Δ*df* = 1, *p* = 0.015, supporting H5 in the hypothesized direction. This finding indicates that family economic investment exerts a stronger driving force on the learning behavior of music majors from rural areas. The visual comparison of key between-group path coefficients is shown in Figure 13.

#### 4.5.3 Crossed multigroup analysis (2 × 2)

To examine whether institution type and student origin jointly shape the family-capital pathways, an additional four-group analysis was conducted using the four crossed cells defined in Section 4.1 (Key + Urban *n* = 281, Key + Rural *n* = 145, Regular + Urban *n* = 266, Regular + Rural *n* = 260). Measurement invariance held from configural through scalar levels across the four groups (configural χ^2^/df = 2.47, CFI = 0.921; metric Δ CFI = −0.006, Δ RMSEA = 0.002; scalar Δ CFI = −0.008, Δ RMSEA = 0.001), supporting valid comparison of structural coefficients.

The constrained-vs.-unconstrained chi-square test for the CC → PSE path across all four cells yielded Δχ^2^(3) = 9.74, *p* = 0.021. The coefficient was highest in the Key + Urban subgroup (β = 0.41) and lowest in the Regular + Rural subgroup (β = 0.22), with Key + Rural (β = 0.35) and Regular + Urban (β = 0.27) falling in between. This gradient suggests a compounded resource advantage: cultural capital is most effective at translating into professional self-efficacy when both institutional and family-environment conditions are favorable.

**Figure 14:**
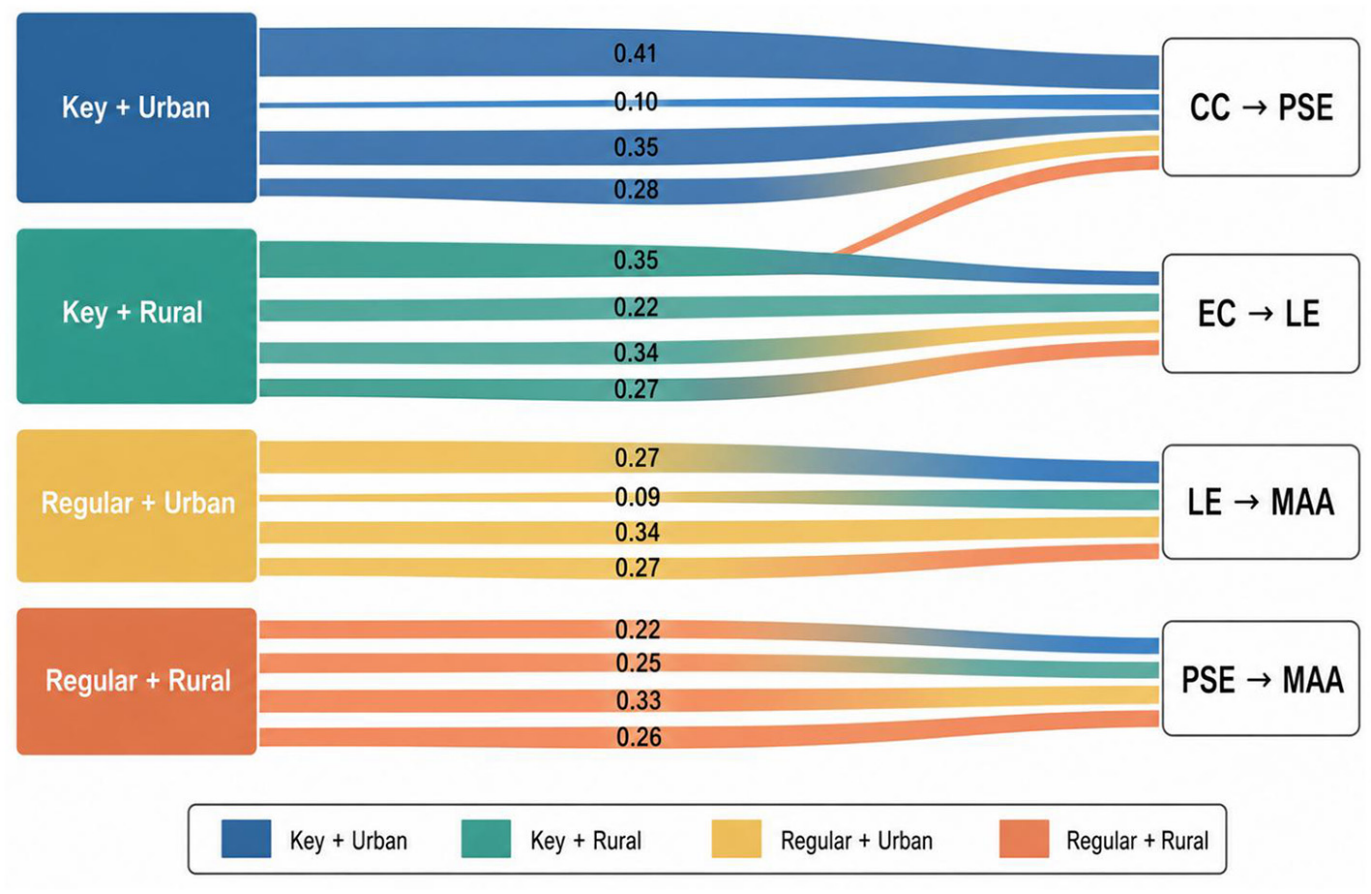
Sankey diagram comparing the panoramic structural coefficients across the four crossed subgroups.

The corresponding test for the EC → LE path yielded Δχ^2^(3) = 8.16, *p* = 0.043, with rural subgroups showing stronger coefficients (Key + Rural β = 0.22, Regular + Rural β = 0.25) than urban subgroups (Key + Urban β = 0.10, Regular + Urban β = 0.09). The crossed pattern is largely attributable to the urban–rural dimension rather than the institutional dimension, supporting the diminishing-marginal-utility interpretation in H5. The complete 2 × 2 comparison is summarized in Table 11. The panoramic structural coefficients across the four crossed subgroups are visualized in Figure 14.

**Table 11:** Standardized path coefficients across the four crossed subgroups.

Path	Key + urban (*n* = 281)	Key + rural (*n* = 145)	Regular + urban (*n* = 266)	Regular + rural (*n* = 260)	*Δχ*^2^(3)	*p*
CC → PSE	0.41	0.35	0.27	0.22	9.74	0.021
EC → LE	0.10	0.22	0.09	0.25	8.16	0.043
LE → MAA	0.35	0.34	0.34	0.33	1.86	0.602
PSE → MAA	0.28	0.27	0.27	0.26	0.92	0.821

The non-significant differences for LE → MAA (Δχ^2^(3) = 1.86, *p* = 0.602) and PSE → MAA (Δχ^2^(3) = 0.92, *p* = 0.821) across the four cells indicate that the conversion of mediators into academic achievement operates as a domain-general process uniform across institutional and geographic conditions. The moderating effects of family background are therefore concentrated at the capital-to-mediator stage rather than the mediator-to-outcome stage—a stage-specific moderation pattern that is consistent with the directional hypotheses derived in Section 3.1 and that refines the moderated-mediation literature.

Based on the above results, of the eleven hypotheses in this study, H1a and H1b were fully supported, H1c was not supported (consistent with the indirect-only mediation classification in Table 9), H2a through H2c and H3a through H3c were all supported, and H4 and H5 received directional support. Cultural capital exhibits the strongest effect across all paths, dominating the direct effect, mediation effects, and the moderated-mediation analyses.

## 5 Discussion and implications

### 5.1 Discussion

This study constructs a multi-path mediation model for the influence of family capital differences on the academic achievement of music majors and systematically tests the data of 952 music majors by structural equation modeling. To clearly present the verification of each hypothesis, all hypothesis testing results are summarized in Table 12.

**Table 12:** Summary of hypothesis testing results.

Hypothesis	Content	Coefficient/effect	*p*/95% CI	Conclusion
H1a	EC has a positive direct effect on MAA	β = 0.12	*p* = 0.004	Supported
H1b	CC has a positive direct effect on MAA	β = 0.19	*p* < 0.001	Supported
H1c	SC has a positive direct effect on MAA	β = 0.08	*p* = 0.060	Not supported
H2a	LE mediates the EC–MAA relationship	IE = 0.051	[0.020, 0.089]	Supported
H2b	LE mediates the CC–MAA relationship	IE = 0.095	[0.052, 0.145]	Supported
H2c	LE mediates the SC–MAA relationship	IE = 0.037	[0.008, 0.074]	Supported
H3a	PSE mediates the EC–MAA relationship	IE = 0.049	[0.021, 0.083]	Supported
H3b	PSE mediates the CC–MAA relationship	IE = 0.084	[0.045, 0.130]	Supported
H3c	PSE mediates the SC–MAA relationship	IE = 0.038	[0.012, 0.070]	Supported
H4 (directional)	CC → PSE is stronger in key universities than in regular universities	Δβ = 0.14 (0.38 vs. 0.24)	Δχ^2^ = 6.82, *p* = 0.009	Supported in direction
H5 (directional)	EC → LE is stronger in rural students than in urban students	Δβ = 0.14 (0.23 vs. 0.09)	Δχ^2^ = 5.93, *p* = 0.015	Supported in direction

As shown in Table 12, eight of the eleven hypotheses received full support, two received directional support, and only H1c was not supported. The results of H1a and H1b indicate that family economic capital and cultural capital can directly promote music majors' multidimensional academic achievement, which is consistent with the positive role of family capital identified in previous basic-education studies (Djekourmane et al., 2025; Jin et al., 2024). At the same time, the applicable boundary of this conclusion is extended to art higher education. The direct effect of cultural capital (β = 0.19) is greater than that of economic capital (β = 0.12). This gap is not accidental: music learning depends heavily on the long-term accumulation of aesthetic literacy and cultural perception, the cultivation of which is far more closely tied to the family cultural atmosphere than to simple economic investment.

Three complementary mechanisms plausibly underlie this domain-specific inversion. The first is the primacy of embodied cultural capital. In Bourdieu's (1984) account, the recognition and appreciation of cultivated musical forms are durable dispositions transmitted through long-term family socialization rather than commodities acquired through short-term expenditure; economic capital can purchase instruments and lessons, but not the accumulated perceptual and interpretive sensibility that performance evaluation rewards. This is consistent with the present data, in which cultural capital exerts the largest effects on both professional self-efficacy (β = 0.31) and learning engagement (β = 0.28). The second is an upstream selection mechanism. Because admission to a Chinese music program is gated by the specialized music entrance examination, which screens applicants on demonstrated skill and aesthetic competence, the enrolled population is pre-filtered toward families that have already supplied embodied cultural capital, so that the variance—and hence the marginal predictive power—of raw economic capital is compressed within the admitted cohort; the range restriction noted in the robustness analysis (Section 4.3) is the empirical footprint of this filtering. The third is the role of cultivated taste as evaluative content. In arts higher education, aesthetic judgment is not merely a background advantage but part of the instructional content and the assessment criteria themselves: the faculty rubric used here explicitly grades musicality and interpretation alongside technical accuracy (Section 3.2.1), so embodied cultural capital maps directly onto the measured achievement construct. These three mechanisms are mutually reinforcing rather than competing; the cross-sectional design cannot adjudicate their relative weight, a task that future work could undertake by incorporating entrance-examination scores and longitudinal admission data.

The non-support of H1c is the most theoretically informative finding in the direct-effect layer. The SC → MAA path estimated within the full structural model that simultaneously contains both mediators is β = 0.08, *p* = 0.060—a non-significant direct effect after the mediation pathways are accounted for. Combined with the significant indirect effects through both learning engagement [IE = 0.037, 95% CI (0.008, 0.074)] and professional self-efficacy [IE = 0.038, 95% CI (0.012, 0.070)], this qualifies as indirect-only (full) mediation under the definition of Zhao et al. (2010) and Hayes (2018): a non-significant direct path together with at least one significant indirect path in the same overall model. The classification of full mediation here is therefore not based on a bivariate test, but on the residual direct path estimated within the full structural model with both mediators present, which is the methodologically rigorous basis for the judgment.

Substantively, this result means that parental music-related social networks and professional information channels do not exert a meaningful direct uplift on music majors' multidimensional academic achievement. Their entire functional contribution operates by motivating student learning behavior and shaping professional self-belief. This finding refines prior work that treated social capital as a generic direct predictor of academic outcomes (Tan and Fang, 2023b), by specifying the behavioral and psychological mechanisms through which social capital is converted into achievement in a high-stakes art-education setting where domain-specific competencies cannot be inherited or transferred directly through parental contacts.

The mediation test results (H2a–H2c and H3a–H3c all supported) constitute one of the core findings of this study. Across all mediation paths, the indirect effect of cultural capital on academic achievement through learning engagement is the largest (IE = 0.095), indicating that parents' music literacy and family cultural atmosphere have the most prominent influence on students' learning behavior—students growing up in families with a music-appreciation tradition are more inclined to invest time in professional practice and to apply more effective practice strategies. The indirect effect of cultural capital through professional self-efficacy is also considerable (IE = 0.084), indicating that students from high cultural capital families develop stronger confidence in their performance ability and stage presence, and this confidence translates into a higher level of academic achievement.

The multigroup comparative analysis provides directional support for the two moderation hypotheses derived from prior theory in Section 3.1 (Whitaker and Wolniak, 2022; Holochwost et al., 2025; Jiang, 2024; Wang and Li, 2024), and the pattern of supported and null moderating effects together carries theoretical weight.

For H4, the path coefficient from cultural capital to professional self-efficacy in key universities (β = 0.38) is significantly higher than in regular universities (β = 0.24), Δχ^2^ = 6.82, *p* = 0.009. This is consistent with the resource-concentration thesis: key institutions provide field conditions (denser professional interactions, more frequent master-class exposure, stronger elite peer effects) that activate the latent aesthetic dispositions inherited from high-cultural-capital families into observable professional self-belief, whereas in regular institutions the activation conditions are weaker. The crossed 2 × 2 analysis (Section 4.5.3) further refines this finding by showing that the activation effect is strongest when both institutional and family conditions are favorable (Key + Urban: β = 0.41) and weakest when both are unfavorable (Regular + Rural: β = 0.22), suggesting a compounded resource-conversion mechanism rather than a simple institutional main effect.

For H5, the path coefficient from economic capital to learning engagement in rural students (β = 0.23) is significantly higher than in urban students (β = 0.09), Δχ^2^ = 5.93, *p* = 0.015. This confirms the diminishing-marginal-utility prediction grounded in Jiang (2024) and Wang and Li (2024): for rural families that operate well below the saturation point of music-related expenditure, each additional unit of professional investment (instrument purchase, private tutoring, performance attendance) produces a substantial behavioral incentive, whereas urban families that already operate near the saturation point obtain limited behavioral returns from additional investment. The crossed analysis confirms that this pattern is driven by the urban–rural dimension rather than by institutional type, since both Key + Rural (β = 0.22) and Regular + Rural (β = 0.25) exceed both urban subgroups (Key + Urban: β = 0.10; Regular + Urban: β = 0.09).

Equally important is what did not differ across groups. The path coefficients from learning engagement to academic achievement and from professional self-efficacy to academic achievement showed no significant variation across either single-grouping analysis (institution type: Δχ^2^ = 1.47, *p* = 0.225; student origin: non-significant) or the crossed 2 × 2 analysis (LE → MAA: Δχ^2^(3) = 1.86, *p* = 0.602; PSE → MAA: Δχ^2^(3) = 0.92, *p* = 0.821). This null pattern is not a methodological limitation but a theoretically meaningful finding: it indicates that once family capital has been translated into engagement and self-efficacy, the conversion of these mediators into academic achievement operates as a domain-general process that is essentially uniform across institutional and geographic conditions. The moderating effects of family background are therefore concentrated at the capital-to-mediator stage rather than the mediator-to-outcome stage. This stage-specific moderation pattern refines the moderated-mediation literature by suggesting that interventions aimed at promoting educational equity should target the activation of family resources (i.e., the formation of engagement and self-efficacy) rather than the latter half of the causal chain, where the conversion mechanism is already equitable across groups.

### 5.2 Implications

The findings of this study provide multi-level references for policy-making and practice improvement in music higher education. At the institutional level, in view of the strongest effect of cultural capital across all paths, music conservatories should focus on building an institutional support system that can bridge the gap between students' family cultural capital. Specifically, by organizing high-frequency master classes, music salons, and art performances, alternative channels can be created for students from low cultural capital families to engage with sophisticated music culture, partly compensating for the inherited deficit of family cultural resources within the school field. The indirect-only mediation pattern for social capital also suggests that universities should attend to the construction of students' professional social networks—building inter-school exchange platforms, establishing alumni mentor systems, and encouraging participation in professional academic communities. These measures can help students with weak social capital obtain more professional development information and support, and indirectly improve their academic performance by enhancing their learning engagement and professional self-belief.

At the differentiated-intervention level, the between-group heterogeneity revealed by the multigroup analysis has a clear policy direction. The amplified driving effect of economic capital on learning engagement among rural students indicates that economic aid policies targeting this group—dedicated scholarships, instrument rental programs, free professional training quotas—can achieve substantial behavioral returns at relatively low cost, exactly because rural families operate well below the saturation point of music-related expenditure. The amplified effect of cultural capital on professional self-efficacy in key universities suggests that regular universities need to invest more deliberately in the cultivation of professional self-confidence during teaching—through expanded performance practice opportunities, more constructive task-level feedback, and a tiered achievement incentive mechanism—in order to narrow the self-efficacy gap caused by differences in institutional field. The core implication is that the realization of educational equity does not rest solely on the equalization of economic resources, but also requires the construction of an institutional cultural environment and the strengthening of psychological support to dissolve the implicit transmission of family capital differences into academic achievement.

## 6 Conclusion

Using 952 Chinese music majors as the sample, this study systematically tested the mechanisms and boundary conditions through which family capital differences affect academic achievement, by means of a multi-path mediation structural equation model. The core conclusions are as follows.

(1) Family cultural capital is the strongest predictor of music majors' multidimensional academic achievement (β = 0.19, *p* < 0.001), and its effect operates through both direct and indirect pathways. The direct effect of economic capital is significant but weaker than that of cultural capital (β = 0.12, *p* = 0.004). The effect of social capital on academic achievement qualifies as indirect-only (full) mediation (Zhao et al., 2010; Hayes, 2018): the direct path estimated within the full structural model containing both mediators is non-significant (β = 0.08, *p* = 0.060), while the indirect paths through learning engagement (IE = 0.037) and professional self-efficacy (IE = 0.038) are both significant.
(2) Learning engagement and professional self-efficacy play significant mediating roles between family capital and academic achievement. All six mediation paths are supported. The indirect effect of cultural capital through learning engagement is the largest (IE = 0.095), followed by the indirect effect of cultural capital through professional self-efficacy (IE = 0.084).
(3) The mechanism of family capital exhibits significant heterogeneity across groups: the effect of cultural capital on professional self-efficacy is stronger in key universities than in regular universities (Δβ = 0.14, *p* = 0.009); the effect of economic capital on learning engagement is stronger among rural students than urban students (Δβ = 0.14, *p* = 0.015). The crossed 2 × 2 multigroup analysis further reveals that moderating effects are concentrated at the capital-to-mediator stage, while the mediator-to-outcome paths (LE → MAA, PSE → MAA) are essentially uniform across institutional and geographic conditions—a stage-specific moderation pattern that refines the moderated-mediation literature.
(4) The multidimensional academic achievement scale for music majors developed in this study (covering performance skills, theoretical achievement, and artistic practice performance) demonstrates good reliability and validity. Performance skills were rated independently by two faculty members per institution using a standardized rubric [ICC (2, *k*) = 0.86]; theoretical achievement was obtained from official course-grade records; and artistic practice performance was composed of competition awards and faculty evaluation. This multi-source measurement design partially decouples the dependent variables from student self-report and substantially reduces common method bias risk by design rather than only by statistical control. The instrument provides a reusable measurement tool for subsequent empirical research in the field of art education.

This study has the following limitations, several of which were partially mitigated by design. First, although the design is cross-sectional and therefore cannot, on its own, establish causal relationships, the risk of reverse causality was reduced through the use of pre-determined family-capital indicators, the fully non-self-report measurement of academic achievement, and the covariate-controlled and reverse-direction robustness checks reported in Section 4.3; residual endogeneity nonetheless remains, and longitudinal tracking or instrumental-variable identification is recommended to confirm the directionality reported here. Second, the sample coverage area is limited; generalization of the conclusions to other countries or cultural settings should be approached with caution. Third, although the dependent variable was measured from non-self-report sources, the independent and mediator variables still rely on self-report; future studies may introduce additional objective evaluation data to further improve measurement precision.

## Data Availability

The original contributions presented in the study are included in the article/supplementary material, further inquiries can be directed to the corresponding author.

## Appendix A. Full Questionnaire items (final version after pilot testing and CFA-based refinement)

All items were rated on a 5-point Likert scale (1 = strongly disagree, 5 = strongly agree), except where indicated otherwise. Two items removed at the CFA stage (annual family income; reverse-scored LE item) are listed in italics for transparency but were not included in the final analysis.

**Table A1:** Economic capital (EC)—three items.

Code	Item
ec1	My family has invested in purchasing or upgrading musical instruments for my professional study.
ec2	My family is able to afford extracurricular professional music training (e.g., one-to-one private lessons, summer camps).
ec3	My family is willing to support my attendance at professional concerts, recitals, or master classes.
*(removed)*	*My family's annual income level is among the upper tier in my hometown*.

**Table A2:** Cultural capital (CC)—five items.

Code	Item
cc1	My parents demonstrate genuine appreciation for music and can discuss musical works with me.
cc2	Listening to or engaging with music is a regular part of family life at home.
cc3	My family owns music-related books, scores, or biographies that I have access to.
cc4	My family owns or subscribes to audio-visual music resources (recordings, streaming, video).
cc5	At least one of my parents has completed higher education.

**Table A3:** Social capital (SC)—three items.

Code	Item
sc1	My parents maintain active communication with my music teachers about my professional development.
sc2	My family knows other families, professionals, or peers connected to music-related circles.
sc3	My parents have multiple channels (professional contacts, alumni networks, online communities) for obtaining information about music education.

**Table A4:** Learning engagement (LE)—five items.

Code	Item
le1	I devote substantial time to instrumental/vocal practice on most days.
le2	I feel genuinely enthusiastic about my music professional studies.
le3	I actively participate in classroom discussions and instructor feedback sessions.
le4	I plan my practice sessions strategically rather than practicing randomly.
le5	I use cognitive strategies (mental rehearsal, self-monitoring, reflective listening) during practice.
*(removed)*	*I rarely think about my musical goals (reverse-scored)*.

**Table A5:** Professional self-efficacy (PSE)—four items.

Code	Item
pse1	I am confident in my ability to perform my main instrument/vocal repertoire to a high standard.
pse2	I am confident in my solfeggio and ear-training abilities.
pse3	I am confident in my ability to master music theory content.
pse4	I am confident in my ability to perform well on stage in front of an audience.

**Table A6:** Multidimensional Academic Achievement (MAA)—three items (non-self-reported).

Code	Source	Description
maa1	Faculty rating (2 faculty members; ICC (2, *k*) = 0.86)	Performance skills, scored on a 10-point standardized rubric covering technical accuracy (4 pts), musicality and interpretation (4 pts), and stage presence (2 pts).
maa2	Official course-grade records	Weighted average score (z-standardized within institution) of music theory and solfeggio courses in the past academic year.
maa3	Award records + faculty evaluation	Composite score of competition awards (institutional/provincial/national) and faculty-evaluated artistic practice performance over the past academic year.

The Chinese version of the questionnaire was developed in parallel through forward-translation, back-translation, and bilingual expert review (Brislin, 1980 procedure). Both language versions are available from the corresponding author upon request.
